# A contribution to the history of the proton channel

**DOI:** 10.1002/wmts.59

**Published:** 2012-08-06

**Authors:** Robert Meech

**Affiliations:** School of Physiology & Pharmacology, University of Bristol, Medical Sciences Building, University WalkBristol BS8 1TD, UK

## Abstract

The low numbers of hydrogen ions in physiological solutions encouraged the assumption that H^+^ currents flowing through conductive pathways would be so small as to be unmeasurable even if theoretically possible. Evidence for an H^+^-based action potential in the luminescent dinoflagellate *Noctiluca* and for an H^+^-conducting channel created by the secretions of the bacterium *Bacillus brevis*, did little to alter this perception. The clear demonstration of H^+^ conduction in molluscan neurons might have provided the breakthrough but the new pathway was without an easily demonstrable function, and escaped general attention. Indeed the extreme measures that must be taken to successfully isolate H^+^ currents meant that it was some years before proton channels were identified in mammalian cells. However, with the general availability of patch-clamp techniques and evidence for an important role in mammalian neutrophils, the stage was set for a series of structure/function studies with the potential to make the proton channel the best understood channel of all. In addition, widespread genomic searches have established that proton channels play important roles in processes ranging from fertilization of the human ovum to the progression of breast cancer. © 2012 WILEY-VCH Verlag GmbH & Co. KGaA, Weinheim.

## INTRODUCTION

By the end of the 1970s secretions collected from the bacterium *Bacillus brevis* had been used to create H^+^ conducting pores in lipid membranes and there was evidence for an H^+^-dependent action potential in light-emitting dinoflagellates. On the other hand the proposed role of proton pores in plant cells remained unconvincing, and it was all too easy to overlook their existence in higher animals. Here I describe how what has come to be called ‘the proton channel’ was uncovered, starting with what we understood about H^+^ conduction at that time.

The process of uncovering the proton channel took place in three phases. The first phase, in the early 1980s, came out of intracellular pH (pH_i_) measurements and voltage-clamp studies on invertebrate neurons.^1^,^2^ The second phase, largely in the 1990s, extended the research to mammalian cells3,4 and the third phase, which exploded in the 2000s, has focused on the cloned channel.5,6 Voltage-gated proton channels are now known to play key roles in organisms as different as phytoplankton^7^ and humans, and in aspects of human physiology ranging from fertilization of the ovum^8^ to the promotion of tumor progression.^9^

In this review I am primarily concerned with phase 1; my coverage of phases 2 and 3 is limited to problems that we found especially puzzling in the early days, such as why proton channels are affected by both calcium and potassium channel inhibitors. In the hope that others may be as entertained as I by interconnectedness and chance, I have set out as clearly as I can the convoluted path that lead to the general realization that H^+^ can travel across all kinds of cell membranes via voltage-gated channels. I have also set out the way in which the technical accomplishments of Roger Thomas and Lou Byerly gave the proton channel field such a firm foundation. I hope to convey the fun and excitement we had doing this work and also what a delight it is to see the latest research starting to explain puzzles we have long wanted to understand.

## NECESSARY BACKGROUND MATERIAL

**Figure 1:**
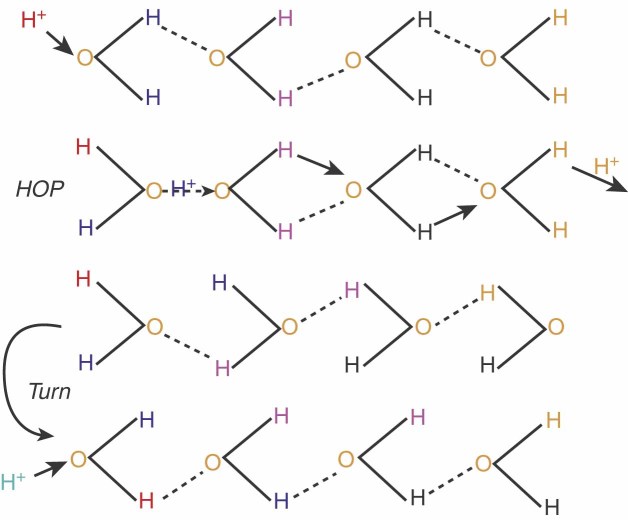
Water wire model to account for the high mobility of H^+^ in water brought about by a ‘Grotthuss mechanism’. Representation of a chain of four water molecules connected by H-bonds. The electrochemical gradient for H^+^ favors their movement from left to right. The approach of an H^+^ (red, top row, left) to the first water molecule in the chain leads to the formation of a covalent OH bond and the partial release of one of its H^+^. This H^+^ (blue, row 2) is shown being shared between water 1 and water 2. Eventually it forms a covalent bond and becomes part of water 2 (blue, row 3). Water 1 turns to its original position, ready to accept a new H^+^ (green, row 4). Addition of each new H^+^ on the left brings about the release a single H^+^ on the right. The intermediate steps are so fast that the H^+^ appears to move rapidly across large distances. (Reprinted with permission from Ref 14. Copyright 2006 Elsevier)

When Lars Onsager delivered his Nobel Prize Lecture on December 11, 1968,^10^ he ended it by describing how an electric current might flow through an ice matrix and he speculated that Na^+^ and K^+^ might pass through biological membranes in much the same way. This speculation turned out to be remarkably fruitful although possibly not quite in the way that Onsager envisioned.^11^ The suggestion was that the amino acid side chains of a membrane protein could form the backbone of a hydrogen bonded network that would be a hydrophilic pathway through the membrane lipid. The simplest network would be made of a single chain of hydrogen bonds commonly called a ‘hydrogen bonded chain’ or sometimes a ‘proton wire’^12^ or a ‘water wire’ (see Figure 1). Such wires are thought to be at the heart of any number of long-range proton transfer reactions including the enzyme, carbonic anhydrase and the transmembrane channel formed by the antibiotic gramicidin. A water wire may be at the heart of the voltage-gated proton channel^13^ that is the subject of this chapter.

This history of the proton channel will make little sense to readers of today unless I explain that there was a time, about 60 years ago, when the only ion channels that anyone knew about were the sodium channel and the potassium channel. I say THE sodium channel and THE potassium channel because so far as anyone knew, there was only one class of sodium channel and one class of potassium channel. And so far as anyone knew their behavior had been described rather completely in the membrane of the squid giant axon, even if they were not identified as channels at the time.^15^ You might think that this was a rather odd situation but sodium and potassium channels satisfied all our needs so far as action potentials were concerned. So although Onsager was aware that H^+^ would travel along a chain of hydrogen bonds he focused his comments on the movement of Na^+^ and K^+^ because it was Na^+^ and K^+^ that were functionally important.

In the 1950s we had no need for other ion channels because even heart action potentials could be explained on the basis of the squid axon formalism.^16^ It is true that there were rumors that crustacean muscle exhibited Ca^2+^-based action potentials but Ca^2+^ was doubly positively charged and would certainly be attached too strongly to the negatively charged cell membrane to be able to travel through any pore-like structure. As Fatt and Katz^17^ commented: ‘The observation that the action potential is retained and, indeed, intensified when the external sodium had been totally replaced by choline is so surprising that we could not help suspecting some error.’ They added: ‘This may seem contrary to the convincing evidence for a direct sodium mechanism which has been obtained in cephalopod axons, and which very probably operates in the same manner in crustacean nerve, but there is no reason to believe that this mechanism is universal.’ In spite of these wise words, the work of Hodgkin and his collaborators was so powerful that the squid axon became a fashionable reference point, almost an institution. So much so that when in 1961 Hagiwara et al.^18^ reported an ‘initial rapid outward surge’ of current upon depolarizing neurons of the snail *Onchidium*, another ten years were to pass before this transient K^+^ current was taken seriously.

The 1970s were boom years for the discovery of new channels. Felix Strumwasser and I^19^ described the Ca^2+^ activated K^+^ conductance, Neher,^20^ Connor and Stevens,^21^ and Gola and Romey^22^ all separately rediscovered Hagiwara's ‘initial rapid outward surge’, or ‘A’-type K^+^ current as it came to be called. Then in 1972 came the evidence for a functioning proton channel. By doping artificial lipid membranes with gramicidin A, a penta-decapeptide antibiotic secreted by *B. brevis*, Myers and Haydon,^23^ demonstrated the creation of narrow conductive pores that could pass alkali metal cations and, more especially, H^+^.

The existence of a conductive pathway for H^+^ had been first proposed in 1968. Studies on giant plant cells such as *Nitella* and *Chara* had shown that their action potentials were based on Cl^−^ currents.^24^ However, at rest the plasmalemma membrane potential in *Nitella* was sensitive to external pH (pH_o_; 50–60 mV per pH unit) and Kitasato^25^ suggested that it had a high permeability to H^+^. Because the membrane potential was 70–80 mV more hyperpolarised than the H^+^ equilibrium potential (*E*_H_; calculated from the pH gradient across the plasmalemma membrane) he proposed that the passive influx of H^+^ was opposed by an outwardly directed ATP-dependent electrogenic pump. Other authors^26^,^27^ accepted the existence of the pump but questioned the presence of the high H^+^ conductance. The problem was that the metabolic inhibitor 2,4-dinitrophenol (DNP) depolarized the membrane to *E*_K_ not the even more positive *E*_H_.^28^ Spanswick also objected to the proposal on functional grounds. Ion transport in plants was thought to be based on a ‘chemiosmotic scheme’, like that in mitochondria,^29^ such that a proton motive force drives the co-transport of Na^+^, anions, sugars, and amino acids. All such transport would be short-circuited by a large passive flux of H^+^. Spanswick suggested that the pH dependence of the plasmalemma membrane potential might come about by a direct effect of the pH gradient on the electrogenicity of the pump.

Mary Rheuben^30^ working in Susumu Hagiwara's laboratory puzzled about an almost identical situation in moth muscle fibers; the resting membrane potential was partially dependent on pH_o_ (20 mV change per unit pH). It was normally more negative than *E*_K_ unless treated with DNP or under anoxia. When she increased membrane resistance by raising external [Ca^2+^] the effect was consistent with the hypothesis ‘that some part of the mechanism maintaining the resting potential was directly dependent upon oxidative metabolism’. Rheuben concluded: ‘The possibility that an H^+^ concentration gradient determines the resting potential cannot be directly excluded at this time; it is considered a less plausible hypothesis on the basis of the low internal pH required and the large fluxes of H^+^ that would occur across the membrane.’

A large transmembrane flux of H^+^ was exactly what was required in the luminescent dinoflagellate *Noctiluca*. If the evidence against an H^+^ conductance in plants was to some extent functional, the evidence for an H^+^ conductance in *Noctiluca* was entirely based on function—at least until 1979. Eckert^31^,^32^ had shown that light emission in *Noctiluca* was associated with an all-or-nothing depolarizing action potential that could be recorded in the thin layer of cytoplasm with the internal vacuolar cavity as reference. Fogel and Hastings^33^ subsequently showed that subcellular particles from another dinoflagellate, *Gonyaulax*, emitted light when the pH of the bathing medium fell from 8 to 5.7. The agent that coupled the action potential with the light emission was an increase in cytoplasmic H^+^; presumably therefore the action potential injected H^+^ into the cytoplasm.^34^

**Figure 2:**
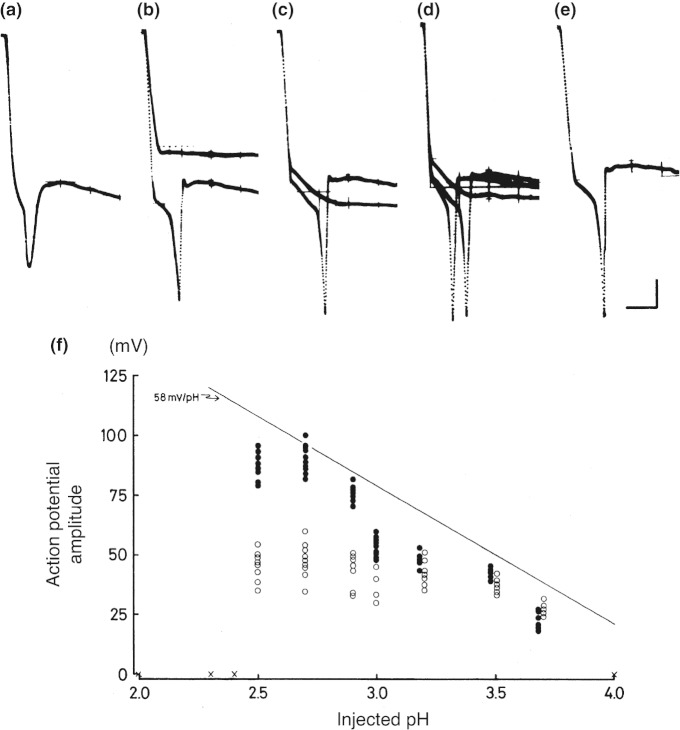
Vacuolar action potential from *Noctiluca* recorded before (a) and after injection of pH 2.7 buffer solution. (b)–(e) were recorded 4, 4.5, 7, and 10 min after start of injection, respectively. Action potentials were recorded across the vacuolar membrane with reference to the external solution and so the action potential is shown as negative-going. Action potentials were evoked by 4.5 × 10^−5^ A/cm^2^ current stimulus, except for a), 4.0 × 10^−5^ A/cm^2^. Experiments were conducted at 21–23°C. Scale bar: 5 ms, 25 mV. (Reprinted with permission from Ref 36. Copyright 1976 Oxford University Press) (f) Relation between the amplitude of flash-associated action potential and vacuolar pH. Action potential amplitude measured 5–15 min after pH buffer injection (closed circles) and after 60 min or more (open circles). The line (58 mV/unit pH) is drawn through the action potential amplitude usually obtained at pH 3.5 (50 mV). Crosses indicate no action potential evoked. (Reprinted with permission from Ref 36. Copyright 1976 Oxford University Press)

The circumstantial evidence for a voltage-gated H^+^ conductance in dinoflagellates would have been persuasive but direct evidence was not available until 1979 when Nawata and Sibaoka^35^ tested the effect of pressure injecting small volumes of different salts and pH buffer (30 mM HCl-glycine) into the vacuolar sap of *Noctiluca* (see Figure 2). None of the major ion species other than H^+^ produced any significant changes in action potential amplitude. Vacuolar sap normally resembles sea water, but has a pH about 3.5.^36^ A vacuolar pH lower than 2.5 damaged the cells irreversibly but the action potential amplitude changed by 58 mV when the vacuolar pH increased from 2.7 to 3.7 (Figure 2(f)) as expected if the vacuolar membrane behaved like an H^+^ electrode. It appeared that during the action potential a transient increase in H^+^ permeability allowed an H^+^ current to flow from the vacuole, into the cytoplasm (assumed to be about pH 7.5 in the resting state).

## HISTORY OF THE VOLTAGE-GATED PROTON CHANNEL IN THREE PHASES

Establishing the existence of ‘the proton channel’ in multicellular animals happened in three distinct phases. I, together with Roger Thomas & Martyn Mahaut-Smith at the University of Bristol and Lou Byerly & Bill Moody at the University of Southern California, were involved with the first phase. This came out of our pH_i_ measurements and voltage-clamp of invertebrate neurons in the early 1980s.1,2,37

Lydia Henderson, also at the University of Bristol and Thomas DeCoursey at Rush University, Chicago spearheaded the second phase. Lydia and her colleagues^3^ thought that voltage-gated proton channels might account for the H^+^ efflux measured during superoxide formation in human neutrophils, but the first direct voltage-clamp evidence in mammalian cells was Tom DeCoursey's paper on rat alveolar epithelial cells.^4^ This work set off a flood of sightings. Henderson's prediction of proton channels in human neutrophils was confirmed in 1993.^38^ In the same year a voltage-dependent H^+^ current was described in cultured human skeletal muscle myotubes^39^ and in the plasma membrane of peritoneal macrophages from mouse.^40^ H^+^ currents are also present in brain macrophages, microglia,^41^ human lymphocytes,^42^ and human granulocytes.^43^ Things progressed so rapidly that in September 2001 Nicolas Demaurex organized the *First* *International Meeting on Proton Channels* in Villars, Switzerland. Shortly thereafter Tom DeCoursey sent me his review: *Voltage-gated proton channels and other proton* *transfer pathways*^44^ which is a superb summary of pretty much everything known about the proton channel at that time and which remains a valuable resource.

The third, most rapidly expanding, phase began properly in 2006 with the cloning of the proton channel by two independent groups.5,6 However, it can be said to have been foreshadowed in 1997 when Starace, Stefani and Bezanilla inserted a histidine residue into the S4 segment of a non-conducting, non-inactivating form of the *Shaker* potassium channel.^45^ The S4 segment, which behaves like the voltage-sensor of the channel, is thought to switch between a ‘gate open’ and ‘gate closed’ configuration. At intermediate voltages, when the sensor would be expected to continuously jump between the two configurations, the histidine-containing mutant appeared to act like a proton shuttle, picking up a proton on one side of the membrane and releasing it on the other. In subsequent work Starace and Bezanilla^46^ were able to create a true proton-conducting pore (later called the ‘gating’ or ‘omega pore’) within the S4 region by substituting an arginine, situated at position 371, with histidine. This work especially interested Lydia Henderson because she had proposed that histidine residues in a putative membrane-spanning region of gp91^phox^ (a component of NADPH oxidase in neutrophils) contributed to H^+^ permeation.^47^

The genome of the marine ascidian *Ciona intestinalis* contributed to the next insight. In a screen for ion channels Okamura and his colleagues^48^ discovered a putative ion channel gene that resembled a phosphatase enzyme. Its full coding sequence appeared to consist of a transmembrane voltage-sensing domain (similar to the S4 segment of *Shaker* and other voltage-gated channels) attached to a phosphatase-like cytoplasmic domain. When expressed in *Xenopus* oocytes this voltage-sensor-containing phosphatase (Ci-VSP) generated transient gating currents but little or no steady current; not surprising considering the absence of any pore-like structure. However, when Okamura et al.^5^ used the amino acid sequence of Ci-VSP to search for other voltage-sensing phosphatases they found a gene that encoded a putative voltage sensor but which lacked any obvious pore or phosphatase domains. This gene was highly conserved and found in tissues ranging from sea urchins to humans. Surprisingly the mouse version which they named mVSOP (mouse voltage-sensor domain-only protein) generated a large voltage-dependent outward current. This current, they found, was carried by H^+^.

In the same year Ramsey et al.^6^ identified a human gene located on chromosome 12 that encoded a 273-amino acid protein (called H_v_1) expressed in immune tissues. When expressed in HEK cells, the protein had the characteristic properties of a voltage-gated H^+^ conductance. Like the *Ciona* VSP, H_v_1 appeared to have charged residues in positions similar to the voltage-sensing domain of the *Shaker* potassium channel.

### Uncovering the Proton Channel; Phase One

My contribution to the proton channel story starts with a series of visits to Harold Mack Brown's laboratory at the University of Utah in the 1970s. ‘Bud’ Brown and I had met as young post-docs at the Scripps Institution of Oceanography, La Jolla, California where Susumu Hagiwara set us the task of establishing the ionic basis of the shadow response in giant photoreceptors of the barnacle, *Balanus* *eburneus*. At that time ‘Hagi’ was thought by many to be the ‘king’ of the calcium channel but his interests were much wider than this title suggests. In 1968 he guided our voltage-clamp study of the light generated current which showed that the mechanism was conductance rather than carrier based.^49^ I eventually moved to Cambridge, England but Mack Brown and I stayed in touch and continued to work together during my brief visits to Utah. It was during one of these visits that we tested the effect on the photoreceptor of 5% CO_2_. The effect was dramatic; a complete, and reversible, abolition of the photo-response. As will become clear, this was the *second step in uncovering the proton* *channel*.

For the first step I should explain why we were using CO_2_ in the first place. The answer is that shortly after my time at La Jolla, I moved north to Caltech to spend a year working with the sea slug *Aplysia californica* in Felix Strumwasser's laboratory. Felix's idea was that I should change the composition of the cytoplasm of one of *Aplysia*'s giant nerve cells. Squid axoplasm could be squeezed out like toothpaste^50^ or digested^51^ and replaced with artificial saline and Felix thought it was time to extend the technique to nerve cell bodies. I began by simply changing the potassium chloride level in the cell and it was only toward the end of 1969 that I found that pressure-injected calcium chloride opened up potassium channels in the cell membrane.^19^ I later called this mechanism ‘calcium-dependent potassium activation’.^52^ Was it possible that Ca^2+^ could open up potassium channels in the cell membrane during normal physiological activity, or was what I had found an artifact brought about by high levels of Ca^2+^ injected under pressure? Having moved again; this time from Caltech to Cambridge, I spent most of the 1970s developing this idea and exploring how the mechanism might work.^53^–^56^

Although taken for granted now, my idea that intracellular Ca^2+^ could activate membrane potassium channels was not accepted in all quarters, and in 1976 my efforts to promote the mechanism received a setback. Observations from Dieter Lux's laboratory in Munich appeared to show that prolonged iontophoretic injection of Ca^2+^ reduced the total net outward K^+^ current in snail neurons.^57^ I too had found that injections of Ca^2+^ could increase membrane resistance but in my hands only with repeated injections of Ca^2+^—never the first time and never when using buffered Ca^2+^. I suspected that mitochondria *in situ* took up 99% of the injected Ca^2+^, just like suspensions of isolated mitochondria, and did so in exchange for H^+^. So I suggested that a high load of Ca^2+^ might make the neuronal cytoplasm markedly acid. Could it be the H^+^ that reduced the outward currents?

First I needed to demonstrate that mitochondrial Ca^2+^/H^+^ exchange occurred *in* *situ* in a single neuron. I could not achieve this myself but I knew a man who could. The *first step in uncovering the proton channel* was when I contacted Roger Thomas who I had known since we were students at the University of Southampton. Roger had published on Ca^2+^ generated proton pulses in suspensions of mitochondria^58^ as well as on pH regulation and buffering in the neurons of the snail, *Helix* *aspersa*.^59^,^60^ It seemed like an ideal partnership and we decided to combine our interests; I injected nerve cells with calcium chloride while he measured their pH_i_ with pH-sensitive glass microelectrodes that he designed and fabricated. We quickly found that for each Ca^2+^ we injected, an H^+^ appeared in the cell cytoplasm.^61^,^62^

The link between steps 1 and 2 is that injection of Ca^2+^ into invertebrate photoreceptors desensitizes them and mimics the process of light adaptation.^63^ Since Ca^2+^ injections acidify the cell cytoplasm, Mack Brown and I wondered whether intracellular acidification produced photoreceptor desensitization. That is why we tested the effect of CO_2_; to raise the levels of carbonic acid in the photoreceptor and acidify its cytoplasm. The dramatic effect we observed led us to suppose that pH_i_ played a role in regulating the photo-response.^64^ The *third step* *in uncovering the proton channel* came from our attempts to link intracellular protons with light adaptation.

Could we show H^+^ build-up in the cytoplasm in the presence of light? Using one of Roger Thomas's pH sensitive microelectrodes (generously provided by Roger himself) we found that the cytoplasm really did go acid in response to prolonged illumination. But where did the acid come from? Assuming it arose from Ca^2+^/H^+^ exchange, did the Ca^2+^ enter the cytoplasm via light-gated channels or was it released from intracellular organelles? Or were there, perhaps, voltage-gated calcium channels in the photoreceptor membrane that opened by depolarization during the receptor potential? We needed to test the effect of light in the absence of depolarization and test the effect of depolarization in the absence of light.

### Voltage-Dependent Changes in Intracellular pH

In January 1979, my wife, young son, and I left Cambridge for a 2-year sabbatical at the University of Utah. Upon arrival in Salt Lake City I set about monitoring pH changes in voltage-clamped photoreceptors. By this time one of Mack Brown's technicians, Toni Gillette, had learned to make ‘Thomas-style’ pH electrodes. This was the *forth step in uncovering the proton* *channel*.

The main difficulty I had with the experiments was that barnacle photoreceptors are covered with a thin but very tough surface layer. Even after treating them with enzyme we had to hammer the recording micropipettes through the cell membrane. It was hard enough to get the two voltage-clamp micropipettes into the cell let alone add a rather blunt pH-sensing electrode. In view of this I decided to test the effect of depolarization on the pH_i_ of a much more amenable cell type, the giant neurons of *H. aspersa*.

To begin with I tested the effect of a relatively modest depolarization to 0 mV. To see any effect at all, I had to hold a neuron at this level for 2–3 min. However, I was delighted to watch as the cytoplasm gradually became more acid. At the time I assumed that the depolarization opened up calcium channels in the cell membrane and the subsequent Ca^2+^ influx was taken up by mitochondria in exchange for H^+^. However, we now know that there is a Ca^2+^/H^+^ exchanger actually in the surface membrane of the neuron^65^ which is the more likely H^+^ source. To try to establish that Ca^2+^ entry was the true source of the acidification I depolarized the neuron nearer to the Ca^2+^ equilibrium potential (E_Ca_). I reasoned that there should be no net Ca^2+^ entry at E_Ca_ and so the closer I got, the slower the acidification should be. To my great surprise, when I depolarized the cell past 0 to +20 or +30 mV the acidification disappeared and the cytoplasm became more and more alkaline; H^+^ appeared to be leaving the cell.

**Figure 3:**
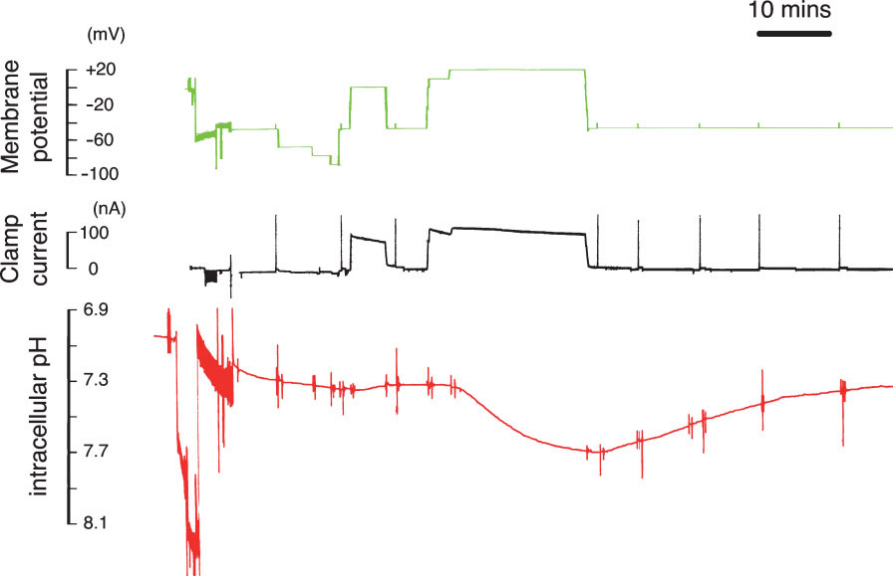
Effect of membrane voltage on pH_i_ in a large neuron from the snail *Helix aspersa*. The cell was penetrated first with a pH-sensitive electrode (bottom trace), then a voltage-recording micropipette (top trace) and finally a current-injecting micropipette (center trace) to voltage-clamp the cell membrane. Periods of hyperpolarization had little or no effect on pH_i_ but a depolarization to 0 mV caused a small acidification with the pH ending up near 7.3. It was not until the membrane was depolarized to +20 mV that anything dramatic happened; then pH_i_ increased rapidly to 7.7. When the membrane was returned to rest (−45 mV) the internal pH gradually returned to near 7.3 (Robert Meech, March 1980, unpublished).

Data from one of my better experiments, dated 14 March 1980, is reproduced in Figure 3. It shows that the cell was penetrated first with a pH-sensitive electrode, then a voltage-recording micropipette and finally a current-injecting micropipette to voltage-clamp the cell membrane. Periods of hyperpolarisation had little or no effect on pH_i_ but a depolarization to 0 mV caused a very small acidification with the pH ending up near 7.3. It was not until I depolarized the membrane to +20 mV that anything dramatic happened; then pH_i_ increased rapidly to 7.7. When I returned the membrane to rest (−45 mV) the internal pH gradually returned to near 7.3.

After a series of these experiments it seemed clear that the cell pH slowly came into equilibrium with the membrane potential and pH_o_. At this stage I had no idea what caused the alkaline shift. It was awkward that the two processes, the Ca^2+^-linked acidification and the alkaline shift, seemed to overlap. To isolate the alkaline shift, I decided to block Ca^2+^ entry (and therefore the acidification) with cobalt chloride. To my huge surprise 10 mM Co^2+^ stopped the alkaline shift dead in its tracks. Could the H^+^ escape through the calcium channels themselves, or perhaps it was the Ca^2+^ activated potassium channels? I had better test the effects of other calcium channel blockers and potassium channel blockers too. I found that the alkaline shift was also blocked by cadmium or zinc ions, or the trivalent ion ruthenium. Even tetraethylammonium ions (TEA^+^) had a slowing effect.

I had read enough about the regulation of pH_i_ to know that the Co^2+^-sensitive, voltage-dependent regulation was an entirely new phenomenon. Library research was so much more difficult then than it is today, but as best as I could I checked back through the literature. As I was looking for examples of voltage-dependent pH regulation I completely missed the *Noctiluca* data.33–36 Instead I found just one short report from the July 1979 *Proceedings of the Physiological Society*.^66^ This showed that external K^+^ appeared to facilitate a cell's recovery from acidification. And who was the author of this report? None other than R.C. Thomas himself! In earlier studies Roger had shown that pH regulation in snail neurons required external Na^+^ and bicarbonate ions for an exchange process that could be inhibited by 4-acetamido-4′-isothiocyanato-stilbene-2,2′-disulphonic acid (SITS). In the Physiological Society report he showed that you could replace external Na^+^ with K^+^ and still get recovery from an acid load—even in the presence of SITS. Perhaps Roger and I were studying the same phenomenon; perhaps the high external K^+^ produced its effect by depolarizing the cell membrane.

By April 1980, I had convinced myself that the alkaline shift was not an artifact and so I phoned Roger to ask if his K^+^-dependent mechanism was blocked by divalent ions. A few days later Roger reported that in isotonic KCl, 1 mM cadmium chloride completely blocked recovery from an acid load. What was even better he then tested the effect of isotonic potassium solution with the cell under voltage-clamp. Now he could acid-load the cell, depolarize it with KCl and then use the voltage-clamp to return the membrane potential to its resting value (−47 mV); the recovery stopped dead.

**Figure 4:**
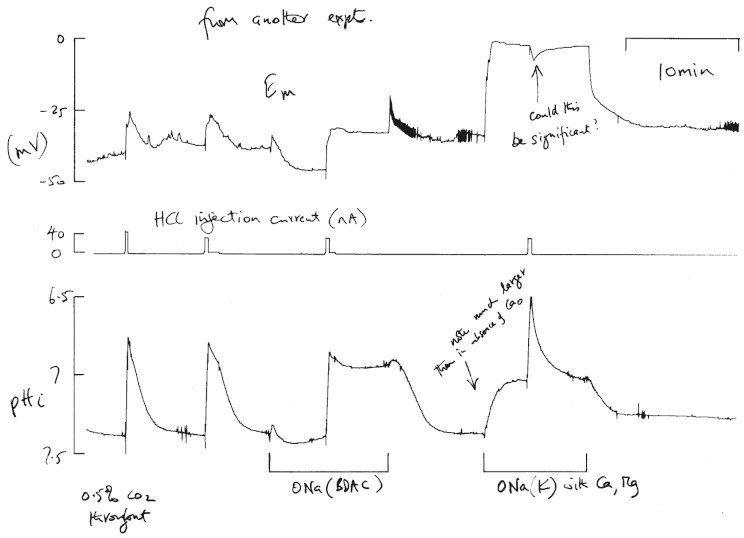
Unpublished experiment by Roger Thomas showing the effects of Na^+^ substitution on the pH_i_ responses to HCl injection into a large snail neuron. In normal saline, iontophoretic injection of HCl (injection current; centre trace) causes a large transient fall in pH_i_ (bottom trace) and a small depolarization of the cell membrane (top trace). Following HCl injection in Na^+^ free (BDAC-substituted) saline there was no pH_i_ recovery until the preparation was returned to normal Na^+^. On the other hand in Na^+^ free (K^+^-substituted) saline the membrane depolarised to near 0 mV and, following HCl injection, pH_i_ rapidly recovered. The injection itself was associated with an increase in membrane potential. 0.5% CO_2_ was present throughout. The hand-written messages to me read: ‘Could this be significant?’ (upper trace) and ‘Note much larger than in the absence of Ca_o_’ (lower trace) (Roger Thomas, April 1979, unpublished).

Shortly after this Roger sent me the record reproduced in Figure 4 ‘from another experiment’. Here, cell pH recovers from iontophoretic HCl injections providing that Na^+^ is present in the bathing medium. Just before the third injection Roger replaced the external sodium chloride with BDAC (bis 2-hydroxyethyl dimethylammonium chloride) showing that recovery was delayed. However, when he replaced sodium chloride with potassium chloride (depolarizing the membrane), recovery was as fast as under control conditions. He makes two written comments, both of which I have retained. The first is that the acidification brought about by the 0 Na (high K) solution is larger in the presence of Ca^2+^ than in its absence (confirming the Ca^2+^/H^+^ exchanger theory); the other indicates the deflection of the membrane potential brought about by the H^+^ injection and asks: ‘Could this be significant?’.

Upon my return to England, the Wellcome Trust facilitated a move from Cambridge to Bristol and promised long-term support. I went house-hunting in Bristol in September 1981, but snatched some of the time to help Roger inject neurons with HCl under voltage-clamp. This experiment required Roger to penetrate a single neuron with five separate electrodes, a real tour de force that eventually provided us with the key evidence. This was the *fifth step in uncovering the proton* *channel*.

The aim of some of our earliest 1981 experiments was to block as much of the outward K^+^ current as possible. There were two reasons for this; one was to improve the voltage-clamp so that we could test the effect of driving the membrane as positive as possible; the other was to explore whether some of the H^+^ current was passing through potassium channels. We started by injecting TEA^+^ but it was not until we used cesium chloride that we saw clear evidence for the H^+^ current.

In our early experiments HCl injections had produced rather variable effects on the outward current and often seemed to reduce it. This may have been a direct effect on the potassium channels themselves and is probably caused by a shift in the voltage-dependence of inactivation as in squid axons.^67^ However, in cesium chloride injected cells at depolarized membrane potentials, recovery after acid injection was clearly associated with an outward current and what is more, it was blocked by low levels of cadmium. Now we had an explanation for the hyperpolarisation in Roger's figure (Figure 4). The finding that recovery was insensitive to metabolic poisons suggested that the pathway was a conductive channel rather than an ATPase and the fact that displacing the membrane potential by 58 mV shifted pH_i_ by a full unit meant that it was a highly selective one. By July 1982 we were ready to send a letter to the journal *Nature*.^2^ The full paper^68^ was delayed by my attempts to determine the rate limiting step for pH recovery; was it diffusion of H^+^ through the cytoplasm or its passage across the cell membrane?

### Residual Currents in Perfused Neurons

My next move was another search of the literature; this time for evidence of H^+^ currents. Again I could find very little. The only possible lead was an odd ‘residual current’ that Lou Byerly and Susumu Hagiwara had found in the neurons of pond snails.^69^ To study Ca^2+^ currents under voltage-clamp without interference from overlapping K^+^ currents, Byerly & Hagiwara had dialyzed the 100 µm diameter neurons by attaching them to a large glass perfusion pipette. When they replaced all the major ions on either side of the cell membrane with large impermeant ones a tiny inconvenient outward current remained. This residual current had an equilibrium potential close to zero mV and appeared to pass through non-selective channels.

Byerly and Hagiwara described how ‘these non-specific currents are sensitive to every treatment that changes the Ca currents. In particular they are strongly suppressed by Ca blockers’. They found that 10 mM external 4-aminopyridine (4-AP), a potassium channel antagonist, slowed down activation and reduced the steady-state current magnitude. In passing, they mentioned that lowering pH_o_ had a similar effect. They said that Doroshenko et al.^70^ had reported much the same in *Helix pomatia* neurons.

I then recalled being invited by Ladislav Tauc to speak about calcium activated potassium channels at one of his *Conferences en neurobiologie de Gif-sur-Yvette*. I remembered the occasion rather well because of the presence not only of Deiter Lux (who was as yet unconvinced that cytoplasmic Ca^2+^ could open potassium channels) but also P.G. Kostyuk from Kiev, who had said that the Ca^2+^ dependent outward current was non-selective. I dug out the papers of Kostyuk and his colleagues Krishtal and Pidoplichko. These authors had used a somewhat different dialysis technique to that of Byerly & Hagiwara but like them had described an outward current, present even when the nerve cells were dialyzed with K^+^-free solution. This ‘Tris current’ was sensitive to cadmium leading Kostyuk to describe it as ‘calcium-dependent’. I was particularly struck by the effect of pH on these currents; there were marked shifts in their conductance curves and changes in their activation time constant and I became convinced that the ‘Tris current’ was almost certainly carried by H^+^.

The *sixth step in uncovering the proton channel* came about when I met Lou Byerly. In August 1982, I was invited to a Gordon Research Conference on Ion Channels in Maine, USA to chair a session on Ca^2+^ activated K^+^ currents. Lou Byerly was another participant and, once our different contributions were out of the way, I remember walking in the sunshine round the quadrangle at Tilton School, near Lars Onsager's ‘country retreat’, describing the experiments that Roger Thomas and I were about to publish. Lou listened patiently to what I had to say about proton channels and residual currents and then suggested that we test the idea together. As luck would have it, a ‘Hagifest’ honoring Susumu Hagiwara's 60th birthday was being planned at UCLA for the following November. ‘Hagi’ was a much-loved teacher and collaborator and the birthday celebration was a very moving affair. It seems appropriate that an event honoring such an innovative neurobiologist should have provided us with an opportunity to discuss and plan new experiments. A photograph in the published proceedings of the meeting^71^ shows Lou and I deep in conversation.

The celebrations being over, we were joined in Lou's laboratory at USC by another of Hagi's protégés, Bill Moody. We had all agreed that the critical test was to perform a ‘tail current’ experiment on a nerve cell bathed in pH 7.4 buffer while perfusing its cytoplasm with cesium aspartate buffered to pH 5.9. Lou and Bill explained that in order to control pH_i_ it would be necessary to use extremely high (i.e. in the order of 100 mM) concentrations of buffer.

**Figure 5:**
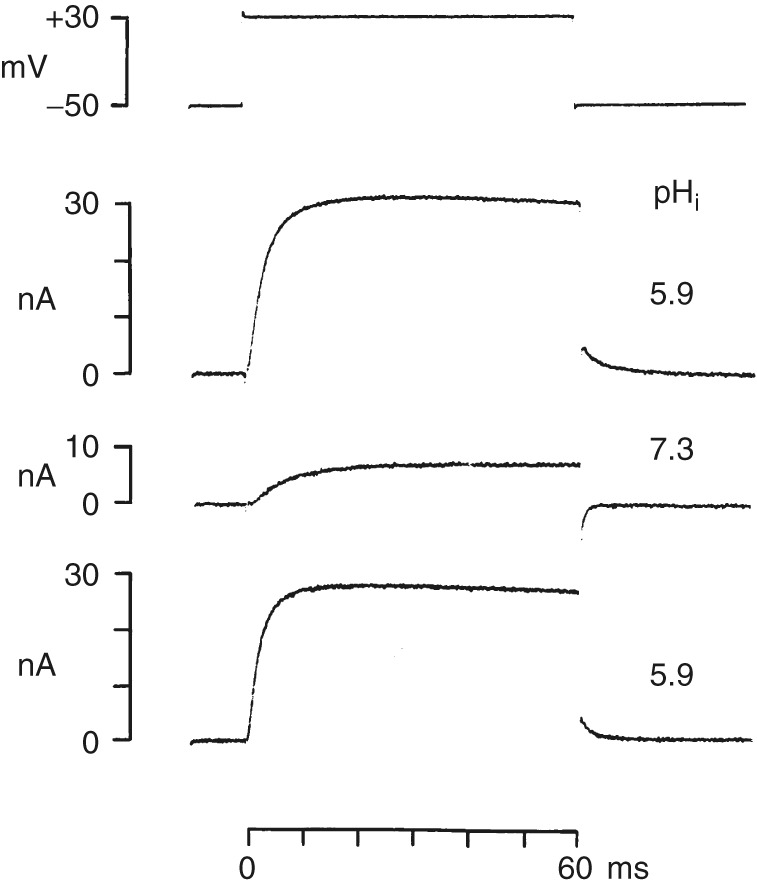
Effect of pH_i_ on the residual current recorded from the pond snail *Lymnaea* *stagnalis*. Membrane currents elicited by an 80 mV step change in the membrane potential (top trace). A tail current is exhibited upon returning the membrane to the holding potential (−50 mV). The pH of the internal solution (pH_i_) is shown on the right; the order of solutions was pH_i_ 5.9 (top), 7.3, 5.9 (bottom). Perfusion time (15 min) was allowed between each solution change to ensure that pH_i_ had reached a stable value.^72^ Internal solution: Cs aspartate, EGTA and MES (pH 5.9) or HEPES (pH 7.3). External solution: hyper-osmotic Tris saline (pH 7.4). Cell diameter, 120 µm; January 1983. (Reprinted with permission from Ref 2. Copyright 1984 The Physiological Society)

The form of the experiment was the same as that set out by Hodgkin and Huxley^15^ in their classic experiments on squid axons. We would first depolarize the cell until we were sure that all the proton channels had been opened; then we would change the membrane potential back near its resting level and watch as the H^+^ current relaxed to zero. Depending on whether the membrane was more positive or more negative than E_H_ this exponentially declining ‘tail’ of current would flow either into or out from the cell. If the membrane potential was set exactly at E_H_, the ‘tail’ would be neither in nor out and we would measure the ‘reversal potential’. Under normal conditions, with a pH of about 7.4 on either side of the membrane this reversal potential would be at 0 mV, but with the cytoplasm as acid as 5.9 the reversal should shift to −87 mV. On the afternoon of the November 11th, 1982, we perfused the cell with the pH 5.9 solution and found that the tail current disappeared when the membrane potential was −67 mV, close enough to the predicted value to reassure me that I was not wasting everyone's time. Figure 5 shows the change in direction of the tail current when pH_i_ was increased from 5.9 to 7.3 and then returned to 5.9.

The apparent simplicity of the experiment as described in the previous paragraph, and the clarity of the result, understates the importance of the work put in by Moody and Byerly to learn how to control pH_i_ in perfused neurons.^72^ They had found that the cytoplasmic buffers were so powerful that the perfused buffer was required to be at an extremely high concentration. Furthermore unless the perfusion pipette had a diameter greater than 30% of the cell diameter, one could have no confidence that the real internal pH was anything like the pH of the perfusion solution.

I returned to Bristol a happy man and waited to hear more from Lou. The first installment of data came in a letter dated, February 24, 1983. In it Lou reported experiments from four cells and listed a series of conclusions. Among them: ‘Outward residual currents are larger in pH_i_ 5.9 than pH_i_ 7.3; voltage dependence appears to be shifted 20–30 mV to the right at pH_i_ 7.3; we never seem to see an inward residual current during the pulse. This is mainly because of the fact that the conductance activation shifts so much with pH_i_. It is reminiscent of the anomalous rectification channel, the activation of which shifts with *E*_K_. Even with pH_i_ 8.2 we only see outward current during the pulse. One way to explain the large voltage shifts with change of pH_i_ and pH_o_ would be to postulate a large negative surface charge at the channel openings; this would also increase the local concentration of H^+^, which would help to explain how such a large H^+^ current can exist in spite of such low bulk concentrations of H^+^’.

**Figure 6:**
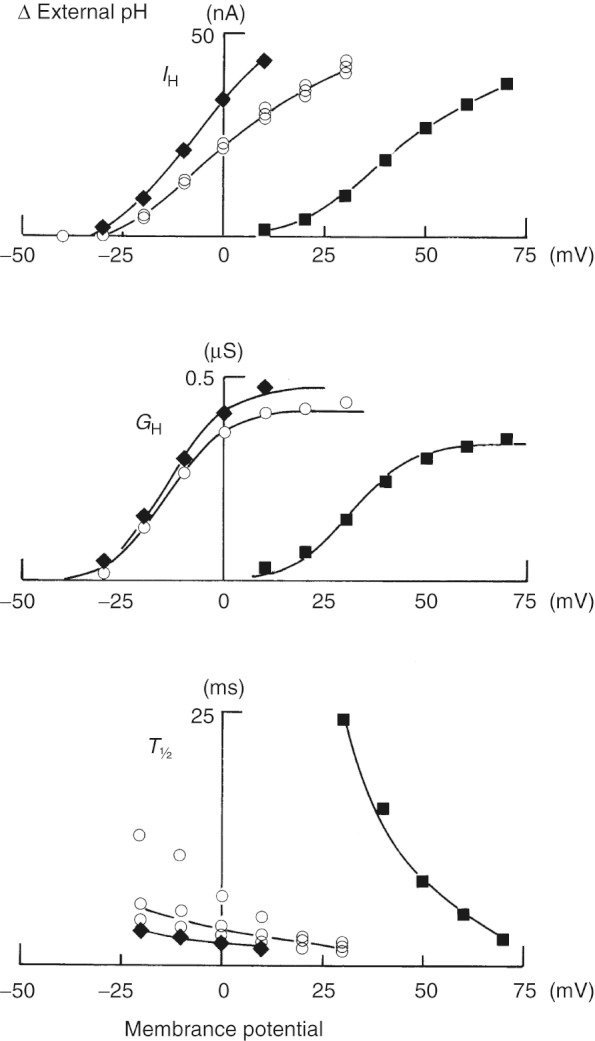
Effect of external pH (pH_o_) on H^+^ current activation. Closed diamonds (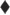), pH_o_ 8.4; open circles (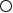), pH_o_ 7.4; closed squares (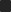), pH_o_ 6.4. The order of solutions was 7.4, 8.4, 7.4, 6.4, 7.4. The currents in the pH 7.4 solution decreased with each change. Internal solution: Cs aspartate, EGTA, MES (pH 5.9). External solution: hyper-osmotic TRIS saline (pH 8.4 and 7.4) and hyper-osmotic TRIS saline with MES (pH 6.4). Holding potential was −50 mV. Abscissa: membrane potential during command pulse. Ordinate, top: I_H_, amplitude of outward current at steady state. Ordinate, center: *G*_H_, H^+^ chord conductance; pH 7.4, final values only. Lines calculated from *G*_H_ = *G*_H,max_/(l + exp[(*V*_H_ − *V*_m_)/7 mV]), where *G*_H,max_ is the maximum H^+^ chord conductance and *V*_H_ is the potential at which *G*_H_ = 0.5*G*_H,max_. *G*_H,max_ assumed to be 0.48 µS, pH_o_ 8.4; 0.42 µS, pH_o_ 7.4; 0.34 µS, pH_o_ 6.4. *V*_H_ assumed to be −14 mV pH_o_ 8.4 and 7.4; +32 mV pH_o_ 6.4. Ordinate, bottom: *T*_1/2_, time to half steady-state current. Note that *T*_1/2_, for pH_o_ 7.4 decreased progressively throughout the experiment. Cell diameter, 100 µm; April 1983. (Reprinted with permission from Ref 2. Copyright 1984 The Physiological Society)

In March the second installment of data arrived in Bristol. Lou wrote: ‘I have sent some data on activation of the H^+^ currents at various external pH's. You didn't ask for the data, but the phenomenon is so striking that I think we should include it in the letter. External pH shifts the gating of the H^+^ current along the voltage axis even more strongly than did internal pH. This is data from only one cell, but we see the effect every time we change external pH. The kinetics seem most strongly affected; a unit decrease in external pH shifts the *T*_1/2_(time for half activation of current) curve to the right by more than 50 mV. This is clearly not a simple screening of surface charge since there is 4 mM Ca^2+^ and 4 mM Mg^2+^ in all external solutions. The strong dependence of gating on both internal and external pH has made it a tricky business to find the reversal potentials.’ The effect of external pH to which Lou was referring is shown in Figure 6.

In the same letter Lou also says: ‘I should comment on how we got rid of Ca currents for these experiments. With pH_i_ = 5.9 there is no Ca current, perhaps due to the large rise in [Ca]_i_ that we have measured during acidification inside the cell. We have done the control experiment of raising [Ca]_i_ while holding pH_i_ constant; there is no change in reversal potential or voltage dependence of activation. Thus the changes are due to changes in pH, not [Ca^2+^]. To measure the H^+^ currents at pH_i_ = 7.3, we first raise [Ca]_i_ until the Ca current disappears, then switch back to low Ca inside. The Ca current does not return (see our abstract in Feb'83, *Biophys* *J*).’

On May 6, Lou reported: ‘I just posted the final grades for the human physiology course I've been teaching *…* So, at last, I'm free to focus on doing experiments again*…*’. He continues: ‘We saw no shift in reversal potential or change in the size of the inward tail current when we changed external buffer strength. So you were right. The size of the currents doesn't appear to depend on buffer strength—a result!’. This was an important result in case the H^+^ current was limited by the amount of mobile buffer. A couple of weeks later I received more data: ‘Cd^2+^ clearly shifts the *I*–*V* and *T*_1/2_–*V* curves to the right. *…*Note that even 10 µM Cd^2+^ has an appreciable effect.’ Finally on May 26: ‘You should have received a total of 5 parcels of data. This is the 5^th^’. ‘At last! I’ve finished all the experiments*…* Well, the ball is in your court now, Bob. I eagerly await the first draft of the paper.’ ‘We should be sure to acknowledge Mr Bruce Yazejian in the paper. He is the graduate student who helped me with most of the experiments.’

Lou finally received the first draft late in July. On August 2 he responded with a revised version of his own, having consulted Bill in Seattle. They were most concerned that we should not push the data too far, as I had a tendency to do. ‘Let's stay away from any modeling of the kinetics of the H^+^ channel. Our clamp is not fast enough to determine if the current turns on truly exponentially; there might be a brief delay.’ ‘Our measurement of inward tail currents is too uncertain to do any reliable extrapolation’. ‘We think that the suggestion that H^+^ currents might go through inactivated fast-inactivating K channels is too speculative to include’.

The paper, which was originally planned as a ‘short *Nature* paper’ that could be written ‘over the Christmas break’ was finally received by the *Journal of* *Physiology* on August 22. Bill and Lou had kept Hagi's UCLA laboratory informed of their progress and on August 31 Bill wrote to say that Michael Barish & Christiane Baud were about to submit their own proton channel paper.^73^ This compared H^+^ currents in immature oocytes of the axolotl *Ambystoma* with our snail data and concluded: ‘As judged by these two preparations, I_H_ seems to be very similar in vertebrate and invertebrate cells’. So no one could say that H^+^ currents were just an oddity seen only in snails.

In October we received an encouraging response from the editor, Philippe Ascher, from his base in Paris: ‘I agree that figures 3 & 4 are quite suggestive that the current is an H^+^ current but in some ways this is so surprising that one cannot exclude some other bizarre interpretations (e.g. the pH_i_ altering the cation selectivity of a channel accepting both Tris and Cs).’ Philippe also questioned whether accumulated ions might dissipate following a pulse producing artifactual tail currents. In addition: ‘I am worried about the size of the current. I have attempted a rough calculation of the single channel current size if the solutions were not buffered and if ion transfer were diffusion limited. I have found 5 × 10^−5^ pA, which leads to an implausible density of proton channels. I understand that in highly buffered solutions like the ones you used the buffer can supply many H^+^ and this may increase the single channel current*…* Would the current be present if the internal solution were not buffered? If so, I think that it would argue against its identification as an H^+^ current’. A referee, also remarked: ‘Nothing is said about *τ*_oﬀ_ (the time constant of the relaxation observed at the end of a depolarizing pulse) although it seems that in many figures *τ*_oﬀ_ was measurable. Did *τ*_oﬀ_ coincide with *τ*_on_ at which both could be measured? The bell shape of the *τ* (V) curve should be mentioned (if not illustrated)’.

On October 31, I wrote thanking Phillippe for his wise comments and said that we had done our best to include his suggested changes. We had included a reference to ‘other bizarre interpretations’, a slightly stronger defense of the tail current measurements and some remarks to the effect that the large H^+^ currents were probably a function of the intracellular buffering capacity but that this was difficult to test in perfused cells because of the inherent buffering capacity of the cytoplasm. I said that our only difficulty concerns the questions about *τ*_oﬀ_. ‘The fact is that we have only really studied the tail currents at potentials about the reversal potential and we have avoided making any comment about the channel kinetics in this paper. This caution is largely derived from Lou's experience with the Ca channel and the knowledge that although the channel appears to have first order kinetics we don't have enough data to be entirely confident*…* We should prefer not to get into this, but we have added a comment to the effect that *τ*_oﬀ_ shifts with pH_i_ and pH_o_ in the same direction as *τ*_on_.’ Five days later we received a note: ‘I like your revised version’.

This is what we published^2^:

‘An appreciable H^+^ conductance might seem unlikely given that the H^+^ activity, internally and externally, is low—even after allowing for the possible increase in H^+^ at the membrane surface caused by the fixed negative charges. But the conclusion that the residual currents are H^+^ currents, is supported by our observations (i) that the tail current reversal potential depends on pH_i_ and pH_o_, (ii) that the maximum conductance for outward currents increases as pH_i_ is lowered and (iii) that the inward tail currents increase in size as pH_o_ is lowered. For these reasons HCO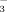, although probably more abundant, is unlikely to be the charge carrier. An alternative hypothesis which is that the selectivity of the channel for cations (Cs^+^ and Tris^+^) against anions (C1^−^ and aspartate) depends on both pH_i_, and pH_o_, is unattractive because of the changes in pH_i_ measured in depolarized *Helix* neurones.^1^ To account for the size of the H^+^ currents we assume (a) that there is a high density of H^+^ channels and/or that, in spite of the low H^+^ activity, there is a large pool of H^+^ available for permeation and (b) that permeation of H^+^ through the channel is high.’

In November Bill and Lou presented a pre-publication report to the Boston Neuroscience Meeting. Lou reported back (letter dated November 17, 1983): ‘Our session was very well attended*…* No one seemed to question our conclusion that these are H^+^ currents; all the questions dealt with the function and other properties of the H^+^ current.’ He added, almost as a post-script: ‘I remembered after talking with you, two experiments I've done that help support my conviction that this current is carried by H^+^ and not Cs^+^, Tris^+^, or Cl^−^. As reported by Byerly and Hagiwara,^69^ replacement of 80% of the external Tris^+^Cl^−^ with glucose does not reduce the ‘nonspecific’ current; so it could not be carried by an influx of Cl^−^. Although not reported, I also tried replacing a large fraction of the internal Cs^+^Asp^−^ with glucose and saw almost no effect on the ‘nonspecific’ current. That result considerably troubled me, since it seemed nothing was left to carry current. So I interpreted the result to mean that the glucose was not getting into the cell (the glucose solution was much more viscous). But after the experiments that Bill and I did on checking the efficiency of internal perfusion, it seems very unlikely to me that it was a perfusion problem.’

All seemed very satisfactory. Now that we knew nerve cells possessed a voltage-gated proton pathway, all we had to do was find out how it worked and what it did. We were to find, however, that this next phase of the story had many unexpected twists and turns.

### Ca^2+^/H^+^ Exchange and a Physiological Function for Proton Channels in Neurons

In 1983 Martyn Mahaut-Smith, who was a graduate student in my laboratory, set about finding a function for proton channels in snail neurons. It seemed possible that they might open during the overshooting phase of the action potential and limit the fall in pH_i_ created by electrical activity.^74^ Our assumption was that the Ca^2+^ influx that took place during a series of action potentials was taken up by mitochondria in exchange for H^+^. Voltage-dependent K^+^ currents were reduced by low pH_i_^75^,^76^ and I knew that an acidification as small as 0.5 pH unit interfered with potassium channel function (unpublished). If we could block proton channels without having too much effect on Ca^2+^ influx we might be able to enhance the fall in pH_i_ and monitor its effects on action potential repolarisation.

**Figure 7:**
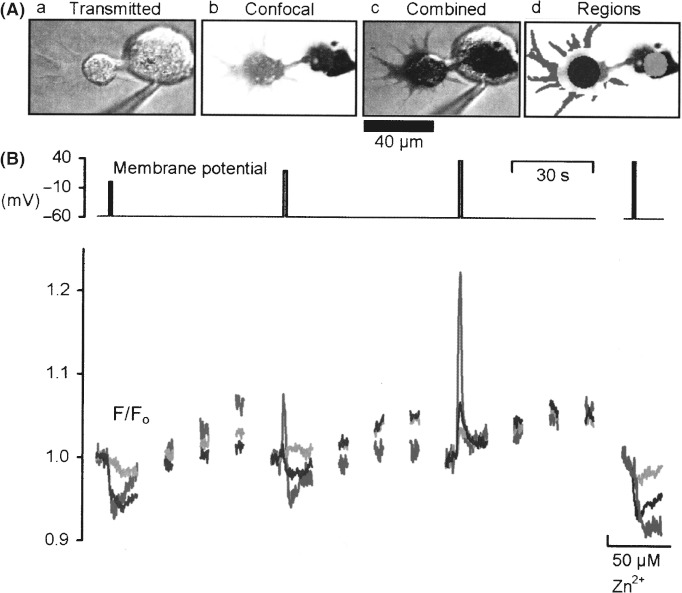
pH-sensitive fluorescence shifts in snail axonal lamellipodia: effect of depolarization and 50 µM Zn^2+^. (Aa) Patch-clamp micropipette attached to neuronal cell soma (transmitted light). (Ab) Confocal image (2.4 µm thick optical slice) showing HPTS fluorescence in the lamellipodia (background subtracted). (Ac) Overlay of images (a) and (b). (Ad) Color coded regions of interest. (B) membrane potential (top record) and relative HPTS fluorescence (*F*/*F*_o_) over time for the three regions shown in (Ad). Absolute fluorescence intensity of lamellipodia (red):axon foot (blue):soma (green) is 13:85:142. Depolarisation (1 second) produces a transient acidification (0 mV; first pulse), a marked alkaline shift (+40 mV; third pulse) or both (+20 mV; second pulse). The alkaline shift is abolished by Zn^2+^. There is a 3 min break in the traces, during which 50 µM Zn^2+^ was applied, before the final depolarization to +40 mV. (Reprinted with permission from Ref 77. Copyright 2002 The Physiological Society)

Martyn found that the peak H^+^ current was reduced by external Zn^2+^ with a *K*_d_ of 16 µmol/L; 1 mM Zn^2+^ would shut down the proton pathway almost completely. Even so we were unable to see any effect on spontaneously active neurons despite 40% of the Ca^2+^ influx being left untouched.^37^ Subsequent experiments by Christof Schwiening and Debbie Willoughby^77^ were rather more successful. Isolated snail neurons readily form flattened extensions of plasma membrane (lamellipodia) that show particularly marked sub-membrane pH changes because of their high surface area to volume ratio. In the presence of 50 µM Zn^2+^ the alkaline shift normally seen in cells depolarized to +40 mV is converted to an acidification (see Figure 7). A similar mechanism operates in neurons from the rat hippocampus.^78^ In both neuronal preparations H^+^ efflux through voltage-gated channels appears to protect the cell from the consequences of Ca^2+^/H^+^ exchange during electrical activity.

Meanwhile Roger Thomas^79^ had found that the outer surface of snail neurons, bathed in weakly buffered saline, became acid when the membrane potential was positive to *E*_H_ and more alkaline with smaller depolarizations. Clearly the pH electrode was picking up the efflux of H^+^ during proton channel activation; more puzzling was how small depolarisations made the surface pH more alkaline. It could not be because H^+^ flowed into the cell because the proton channel was generally closed to H^+^ influx. The explanation turned out to be that Ca^2+^/H^+^ exchange takes place across the cell membrane itself^65^ (where it is better known as the plasma membrane Ca^2+^ ATPase; PMCA). A small depolarization would raise the level of internal Ca^2+^ which was exchanged for external H^+^, increasing the pH of the external surface. Ca^2+^ injection produced an external alkaline shift by the same mechanism. Vanadate injection, which inhibited Ca^2+^/H^+^ exchange, blocked the alkaline shift in each case.

Long before the true nature of the ‘residual current’ had been identified at least two different groups had explored the possibility that it might contaminate Ca^2+^ current measurements.^69^,^80^ In Kostyuk's laboratory, Doroshenko et al.^81^ showed that Ca^2+^ current inactivation could be described by the sum of two exponentials but that the faster component could be eliminated by raising pH_i_ to 8.1. If fast ‘inactivation’ was actually an outward H^+^ current in disguise, it might contribute to membrane repolarization as well as minimize cell acidification. Curiously, the fast component was also abolished by extracellular Ba^2+^ showing that the H^+^ current contribution to ‘fast inactivation’ does not arise from depolarization alone.^82^ Roger Thomas now finds^83^ that Ba^2+^ exchanges with H^+^ at the surface membrane just as Ca^2+^ does, albeit rather slowly. As a consequence, in the presence of external Ba^2+^ any H^+^ current will be delayed and mixed in with the slow phase of inactivation.

### Uncovering the Proton Channel; Phase Two, Mammalian Cells

If I had expected that proton channels in an obscure mollusc would evoke an excited world-wide reaction, I would have been greatly disappointed. Eventually in 1987, Lydia Henderson and her colleagues^3^ identified a possible role for a gated H^+^ conductance in mammalian immune cells. The idea was that proton channels might rid neutrophils of unwanted H^+^ built up as a by-product during phagocytosis. Neutrophils kill bacteria and other invading cells by showering them with the highly reactive oxygen species, superoxide (O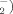, a process that liberates two H^+^ into the neutrophil cytoplasm. Were it not for some compensatory mechanism, neutrophil pH_i_ would fall precipitously and the membrane potential would become large and positive. These side-effects would limit the amount of superoxide generated unless the depolarization activated an H^+^ conductance allowing the unwanted H^+^ to escape the cell.

Direct evidence for a voltage-gated H^+^ conductance in mammalian cells came later. In February 1991, at the annual meeting of the Biophysical Society in San Francisco, Thomas DeCoursey^84^ reported an H^+^ current in cultured alveolar epithelial cells. Using the whole-cell patch-clamp technique with impermeant ions on either side of the cell membrane and a pipette solution buffered to pH 5.5, he showed tail currents that changed as expected for H^+^ selectivity. The H^+^ currents activated and deactivated more slowly (full activation takes seconds) than the ones we had recorded in snail (where the time to half activation was as little as 1 ms) but just like the snail currents they were inhibited by low levels of Cd^2+^ (10–100 µM). Two years later DeCoursey and Cherny^38^ found H^+^ currents in human neutrophils. Perhaps proton channels were of general interest after all!

### Effect of pH_i_ and pH_o_ on Proton Channel Gating in Snails and Mammals

Cherny et al.^85^ found that in mammalian cells shifts in the voltage dependence of activation were surprisingly similar whether one changed pH_i_ or pH_o_. They proposed that proton channel gating depended on the pH gradient across the cell membrane. DeCoursey^44^ has gathered a mass of data, to show that the relationship between gating and the pH gradient can be defined by the equation:


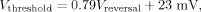
(1)

where *V*_reversal_ is measured during tail current experiments and *V*_threshold_ is the voltage at which H^+^ currents first appear, typically ∼1% of the maximum H^+^ conductance.^86^ This approach avoids the question of whether pH_i_ at the cell membrane is the same as in the perfusing solution and it brings out rather nicely the fact that when there is no pH gradient across the cell membrane (i.e. *V*_reversal_ is 0 mV) the threshold for H^+^ currents is always more depolarized than the reversal potential. Just as in snail neurons, proton channels function in mammalian cells to ensure that H^+^ current is always outward.

It is noteworthy that the corresponding relationship for kH_v_1, a proton channel identified from cDNA from the dinoflagellate *Karlodinium veneficum*, has a *V*_threshold_ offset 37 mV negative to *V*_reversal_.^86^ This means that a stimulus that takes the membrane to threshold could generate a regenerative H^+^-based action potential. *Karlodinium* is not a light-emitting species but it is possible that trans-vacuolar membrane action potentials contribute to the release of karlotoxins during algal blooms. If proton channels in *Noctiluca* behave in the same way, a flux of H^+^ from the vacuole could raise the H^+^ concentration in the thin layer of cytoplasm and provoke the emission of light. The fall in pH will also shift *V*_threshold_ and reduce the channel's open probability so that the vacuolar membrane will tend to return to its resting value. Although a contributory factor to action potential recovery it is probably not the only one as recovery takes place in two phases (see Figure 2(a)) and is prolonged by injections of KCl into the vacuole, a hint perhaps at the involvement of voltage-gated potassium channels.

As Lou Byerly's March 1983 letter makes clear, the *Lymnaea* experiments showed that pH_o_ changes produced rather greater shifts in activation than changes in pH_i_. I have reevaluated our published data^2^ by adopting DeCoursey's method of plotting the threshold voltage for the appearance of H^+^ current against the H^+^ current reversal potential. To adapt equation 1 to the *Lymnaea* data the slope factor must be reduced to 0.5 for changes in pH_i_, and the threshold must be offset by about 8 mV instead of 23 mV. For changes in pH_o_ we find that the slope factor is much steeper (1.35) and the offset is more positive (60 mV). This is shown in Figure 9(a) together with data from Doroshenko et al. for pH_i_ changes in *Helix* neurons.^81^

Before leaving this comparison between mammalian and molluscan proton channels I must note that there were differences in recording conditions. In our *Lymnaea* experiments^2^ the major internal cation was Cs^+^ while the major external cation was Tris^+^. Doroshenko et al.^81^ used Tris^+^ as the major cation inside and out in their experiments. In alveolar epithelial cells Cherny et al.^85^ used tetramethylammonium ions (TMA^+^) as the major cation, both inside and out. (They also added EGTA to external solutions). Although it is conceivable that TMA^+^ has a less ‘disruptive’ effect on the ice-like structure of water than either Cs^+^ or Tris^+^ it is hard to see how this would translate into the differences observed.

In the early 1980s the prevailing view was that shifts in channel gating, were probably the result of interactions between ions and membrane surface charges. In one version of the theory, a proportion of the lipid in the cell membrane was assumed to have a net negative charge. If each lipid molecule takes up about 60 Å of membrane surface,^87^ the bilayer will have one electronic charge/60 Å. Such charges will develop an electrostatic force which will be balanced by a diffuse layer consisting predominantly of cations. The interaction between the cations and the membrane surface may consist of non-specific ‘screening’ of the negative charges or specific ‘binding’; the difference stemming from the closeness of the interaction. During screening there is no change in the density of the surface charges because the interaction takes place at a distance; during binding the nature of the cation becomes important because of the closeness of the interaction. I give a more complete list of references in a review published in 1986.^88^

In *Lymnaea* neurons proton channel activation shifts by about 20 mV when pH_i_ goes from 7.3 to 5.9. To explain this, the fixed charge hypothesis required the charges to be separated by no more than 30 Å,^89^ which seemed just possible. The shifts in activation produced by changes in pH_o_ (46 mV per unit pH change), however, made the surface charge explanation rather less likely. We were therefore attracted to a proposal by Gilly and Armstrong^90^ designed to account for the effect of Zn^2+^ on Na^+^ activation in squid axons. The essential feature of this model was that divalent ions might interact directly with the channel gate.^2^ It follows from the theoretical treatment set out by Hodgkin and Huxley,^91^ that 50% channel activation occurs at the voltage where the rate constant for channel opening equals the rate constant for channel closing. Consequently if H^+^ alters channel opening alone, channel closing alone or has differential effects on both channel opening and channel closing the activation curve will shift along the voltage axis.^88^ Cherny et al.^85^ have suggested that a binding site accessible to external H^+^ must be deprotonated before the channel can open; opening causes the site to ‘disappear’, the same site, or an equivalent, reappearing on the internal side of the membrane. If the same site interacts with both internal and external H^+^ this would account for the findings in mammalian cells. For snail cells the H^+^ binding properties of the site would have to be influenced by its changing local environment.

## DISCUSSION

### Effect of 4-Aminopyridine

Lou Byerly and Susumu Hagiwara had long used 4-aminopyridine (4-AP) to minimize the ‘residual current’ that persistently interfered with their calcium current studies.^69^ In the subsequent ‘proton current’ paper we found that 10 mM 4-AP would reduce the maximum H^+^conductance to a third of its control value.^2^ It did this with little or no change in the voltage-dependence of activation although there was a marked slowing in the rate of rise of the current. This effect remains something of a puzzle. The problem is that 4-AP is a weak base that can diffuse across the cell membrane, ionize and increase pH_i_. Thomas and I later reported^68^ that 10 mM 4-AP not only increased pH_i_ but also raised the internal buffering power of the cell. This made us unsure whether or not 4-AP had a direct effect on the H^+^conductance.^68^ However, in perfused *Lymnaea* neurons the interpretation is more straightforward; any increase in pH_i_ should cause a marked shift in the voltage-dependence of proton channel activation. The *Lymnaea* H^+^ currents displayed no activation shift and so it is safe to assume that 4-AP does directly reduce the H^+^conductance. 4-AP also reduces H^+^currents in other preparations, such as microglia.^92^

The significance of the 4-AP observations remained obscure until the discovery that H_v_1 strongly resembled a potassium channel voltage-sensing domain. In 2001 Armstrong and Loboda^93^,^94^ had presented a model for the action of 4-AP on potassium channel gating. As with all pharmacological agents 4-AP action depends upon its gaining access to an active site. In this case the active site is deep within the pore of the potassium channel and so 4-AP only acts on open potassium channels. For our purposes this is less interesting than what 4-AP does when it gets into the channel. Armstrong & Loboda^94^ say: ‘Once in the open channel, 4-AP's major action is to bias the activation gate toward the closed conformation by approximately the energy of a hydrogen bond. S4 segment movement, as reflected in gating currents, is almost normal for a 4-AP-occupied channel; only the final opening transition is affected.’ In the proton channel the active site is not hidden in a gated pore and it is attractive to suppose that 4-AP acts directly on S4, stabilizing it so that the H^+^conducting pathway remains incomplete.

### Effect of Divalent Ions

**Figure 8:**
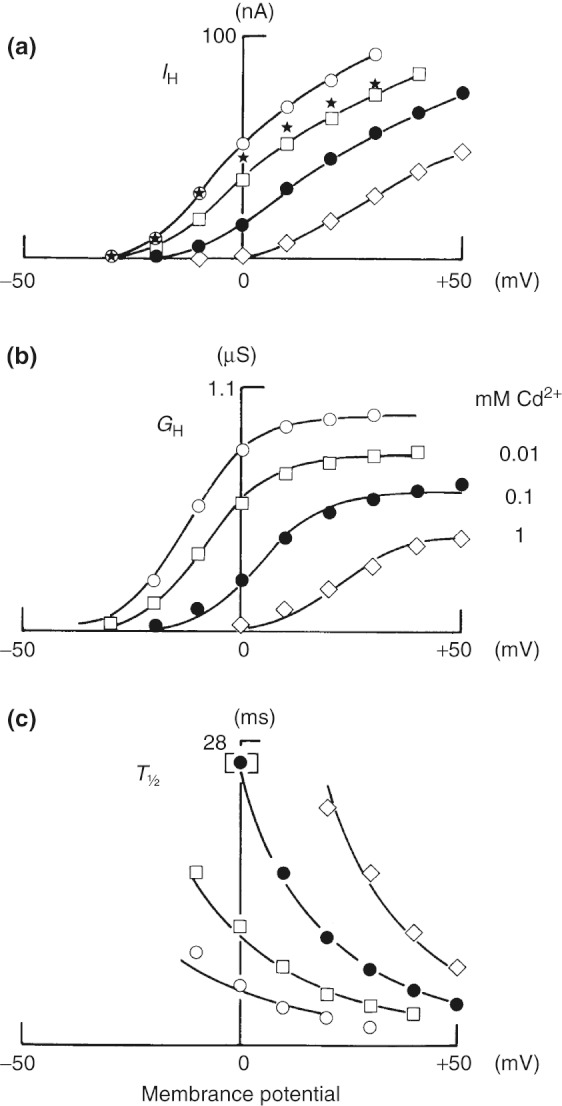
Effect of external Cd^2+^ on H^+^ activation. Open circles (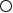), initial control; open squares (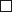), 0.01 mM Cd^2+^; closed circles (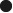), 0.1 mM Cd^2+^; open diamonds (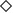), 1 mM Cd^2+^; stars (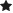), final control. Internal solution: Cs aspartate, EGTA, MES (pH 5.9). External solution: hyper-osmotic TRIS saline (pH 7.4). Holding potential was −50 mV. Abscissa: membrane potential during command pulse. (a) Ordinate: *I*_H_, amplitude of the outward current. (b) Ordinate: *G*_H_, H^+^ chord conductance. Lines fitted to the experimental results determined from *G*_H_ = *G*_H,max_/(l + exp[(*V*_H_ − *V*_m_)/7 mV]), where *G*_H,max_ is the maximum H^+^ chord conductance and *V*_H_ is the potential at which *G*_H_ = 0.5*G*_H,max_. *G*_H,max_ assumed to be 0.98 µS, 0 Cd^2+^; 0.80 µS, 0.01 mM Cd^2+^; 0.64 µS, 0.1 mM Cd^2+^; 044 µS, 1 mM Cd^2+^. *V*_H_ assumed to be −12 mV, 0 Cd^2+^; −7.5 mV, 0.01 mM Cd^2+^; +5 mV, 0.1 mM Cd^2+^; +22.5 mV, 1mM Cd^2+^. (c) Ordinate: *T*_1/2_ time to half steady-state current. The line through the 0.1 mM Cd^2+^ experimental points was drawn by eye; other lines drawn by assuming shifts of +20, −20, and −40 mV along the voltage axis. Note: the point enclosed by the square brackets is an approximate value. Cell diameter, 120 µm; April 1983. (Reprinted with permission from Ref 2. Copyright 1984 The Physiological Society)

**Figure 9:**
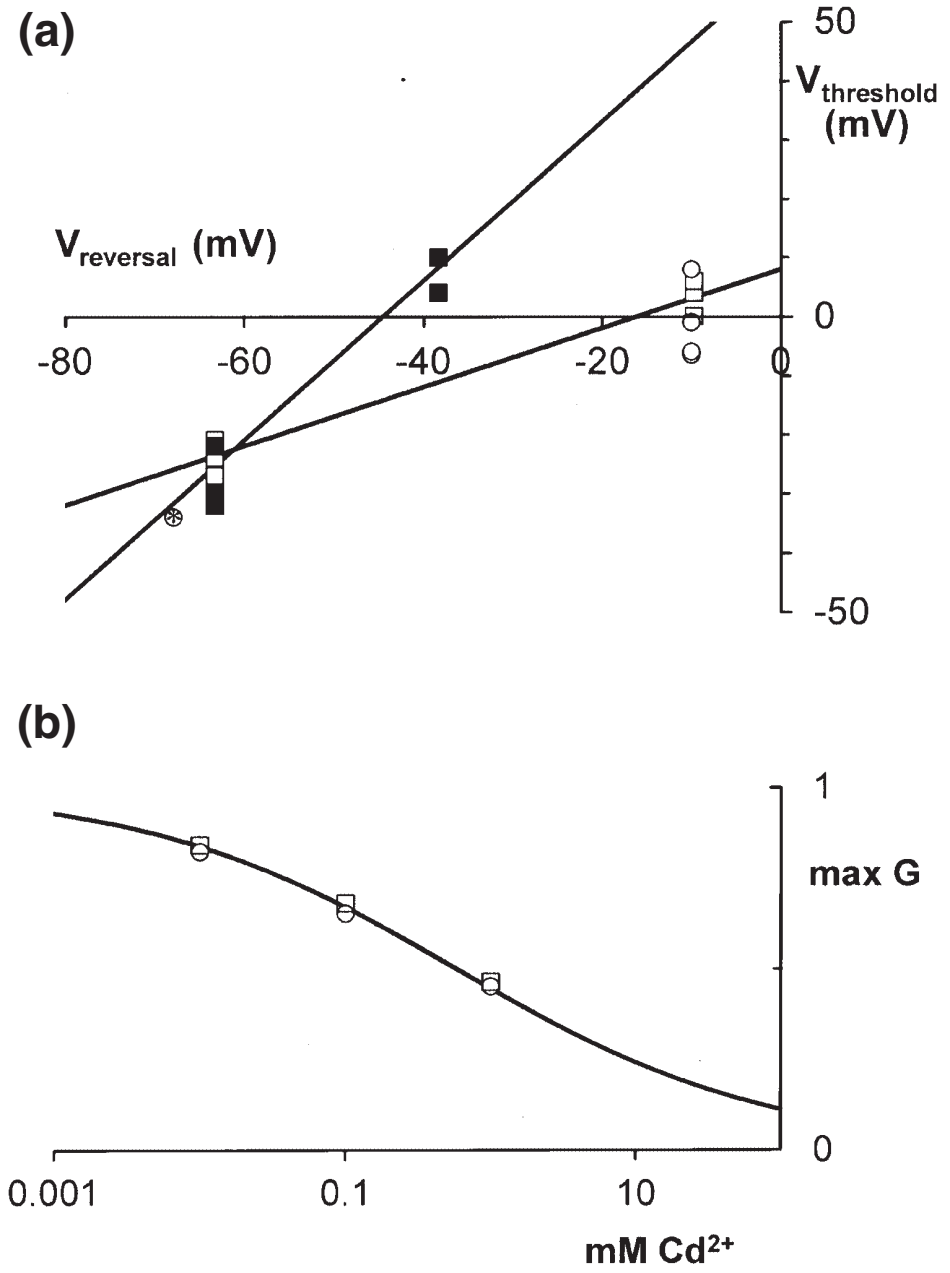
Proton currents in molluscan neurons. (a) Effect of changes in pH_i_ (open symbols; squares (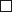), pH_o_ 7.4; circles (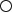) pH_o_ 7.5), and pH_o_ (filled symbols; squares (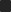), pH_i_ 5.9) on the activation threshold (*V*_threshold_; ordinate). Changes in pH were followed by measuring the tail current reversal potential (*V*_reversal_; abscissa). Square symbols from data in Byerly et al.^2^; circles from data in Doroshenko et al.^81^ Asterisk (*) indicates extrapolated *V*_reversal_ for pH_i_ 5.8. Lines drawn through the points as explained in text. (b) Effect of external Cd^2+^ on the maximum proton conductance. Different symbols show data from different cells. The line drawn through the points is a Hill plot; slope 0.4; K_d_ 0.8 mM. (Data replotted from Ref 2)

Another important feature of the *Lymnaea* proton channel is its interaction with Cd^2+^; as Figure 8 shows the shift in the time to half-activation is quite marked, but so too is the effect on maximum conductance. In alveolar epithelial cells on the other hand, almost all of the effects observed can be attributed to a shift in the activation curve with relatively little effect on the maximum conductance.^95^ In Figure 9(b) the *Lymnaea* data^2^ is replotted to show the effect of Cd^2+^ on the maximum conductance alone (i.e. separated from any shift in activation). The line through the data is a Hill plot with a slope of 0.4 and a K_d_ of 0.8 mM. By contrast 0.1 mM Cd^2+^produces no block at any voltage in alveolar epithelial cells and even at 10 mM there is only 20% inhibition. The shift in voltage dependence is also less than that in *Lymnaea*: in alveolar epithelial cells the shifts for 0.1, 1.0, and 10 mM Cd^2+^ are 0, 8, and 34 mV while in *Lymnaea* the shifts for 0.01, 0.1 and 1 mM Cd^2+^ are 6–10, 16–25, and 32–48 mV. In *Ambystoma oocytes*,^73^ H^+^ currents are also shifted and blocked by low levels of Cd^2+^ and in other mammalian cells, such as granulocytic HL-60 cells, 0.15 mM Cd^2+^ reduces the maximal conductance by 26% while shifting the conductance–voltage relationship by 40 mV.^43^

Recent studies^96^–^98^ show that heterologously expressed human (H_v_1) and mouse (mVSOP) voltage-gated proton channels exist as dimers. Furthermore dimers consisting of a wild type-histidine substituted (H140A/H193A) tandem are less shifted by Zn^2+^ suggesting that both partners contribute histidines to the divalent ion binding site.^99^ It is tempting to imagine that the 0.4 slope of the Hill plot (Figure 9(b)) of the data we obtained for the Cd^2+^ block of proton channels in *Lymnaea* is also a reflection of their dimeric nature.

### Mechanism of Proton Permeation

After a successful experiment in Lou Byerly's laboratory we would speculate about how H^+^ might pass along or through the proton channel. One idea was that they were conducted along the anionic head groups of the lipid bilayer.^100^ Effectively the membrane would act as an aerial (‘antenna’), collecting H^+^ whenever they collided with the membrane surface and funneling them to the channel. Reducing the number of dimensions from three to two can reduce diffusion times providing that the forces holding the H^+^ to the surface are sufficiently weak.^101^ As for the proton channel itself, we wondered whether H^+^ could travel through fissures between membrane proteins and their lipid environment.

Although H^+^ currents appeared to activate and deactivate like any other voltage-gated current, we did not really know whether the proton pathway was a carrier or a channel. In 1985, Lou Byerly wrote to suggest we join forces in a hunt for single channel H^+^ currents in order to better define the mechanism. Unfortunately a visit to Los Angeles was not practicable at that time and Lou eventually recruited Yu Suen. Their studies^102^ on inside-out membrane patches showed that K^+^ currents and H^+^ currents were distributed quite differently in the cell membrane. While 85% of all patches had K^+^ currents, only five out of 38 patches (i.e. 13%) had H^+^ currents. Patches with H^+^ currents showed no sign of individual channel openings and the data suggested an upper limit for the unitary current of 4 × 10^−3^ pA at +10 mV. The maximum H^+^ current density was 29 nA/nF which translates into about 50 channels/µ^2^(assuming the upper limit for the unitary conductance and a membrane capacitance of 1 µF/cm^2^). However, if the channels were concentrated into 13% of the cell surface, channel densities could exceed 400 channels/µ^2^in isolated clusters.

Our speculations about how H^+^ moved along the proton pathway needed all the constraints that good experiments can provide. Byerly and Suen^102^ set about establishing these constraints by measuring the temperature dependence of H^+^ permeation. Currents carried by ions passing through an open water-filled pore should be limited by rates of diffusion and the temperature dependence of a physical process of this kind would be expected to be low. The 10° temperature coefficient (*Q*_10_) for diffusion of an ion depends not only on its kinetic energy (linked to the thermodynamic ‘absolute’ temperature; i.e. *Q*_10_ of 1.03–1.04 at 20°C) but also on the fluidity of the medium through which it passes (*Q*_10_ for water about 1.3 at 20°C). For a chemical reaction the temperature dependence is higher than this because the process depends on interactions that cannot proceed unless the reactants have attained their ‘activation energy’. Byerly and Suen^102^ compared the *Q*_10_'s of H^+^ and K^+^ currents in the same membrane over the same temperature range (10–24°C); the *Q*_l0_ of the H^+^ current was 2.1 ± 0 4, while that of the K^+^ current was 1.4 ± 0 04. A value above 2 meant that if the pathway was a pore, someway along its length there was a significant barrier to H^+^ passage. At one extreme it might simply represent an unfavorable location faced by the H^+^ as it ‘hops’ from one favorable site to the next; alternatively H^+^ transfer may require a fully fledged conformation change. Broadly speaking the greater the change in pathway conformation the greater the expected temperature dependence.

In a detailed analysis of whole cell experiments on a variety of different mammalian cell types DeCoursey and Cherny^103^ found proton channel *Q*_10_'s to range from 2.1 to 3.1. For inside/out membrane patches the value was 2.8 increasing to 5.3 below 20°C. In whole cells above 30°C the *Q*_10_ was unusually low probably because of the depletion of H^+^. Depletion was considered to be less likely in detached patches because the volume of the bathing solutions were ‘effectively infinite’.

As expressed by Peter Lauger:^104^ ‘If the permeability of an ion channel is high, the overall transport becomes ultimately limited by the rate with which ions from the aqueous phase arrive at the mouth of the channel’, i.e. the permeability of the pore would depend not only on its intrinsic permeability but also on the permeabilities of the intracellular and extracellular convergence pathways. The conductance of each convergence pathway would depend on the capture radius of the pore and the diffusion coefficient of the moving ion, this latter being proportional to the fluidity of the solvent (inverse of viscosity). Andersen,^105^ who examined saturating currents flowing through gramicidin A channels, added sucrose to the bathing medium to show that the limiting conductance of the channel decreased as the viscosity of the medium increased.

**Figure 10:**
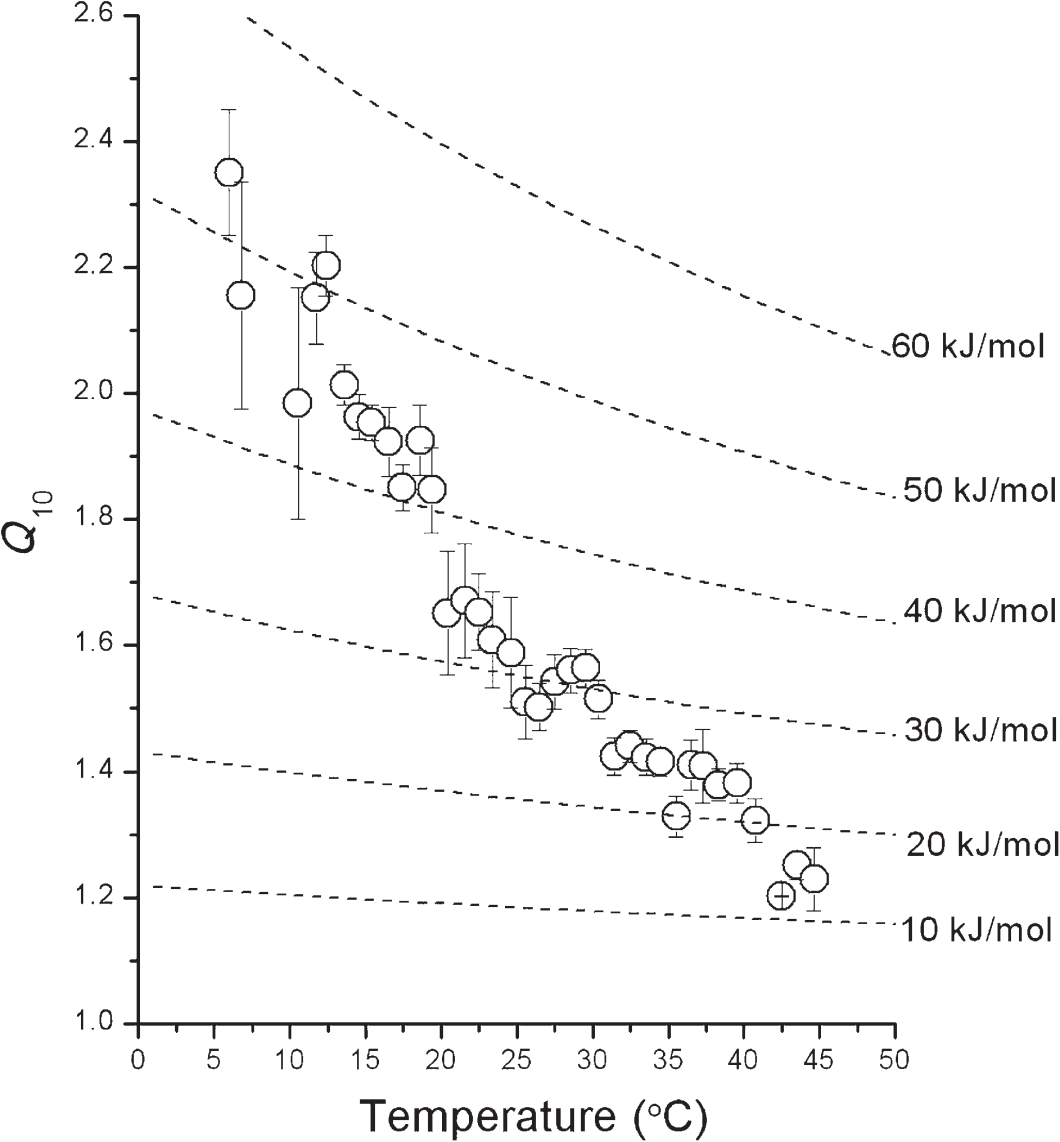
Temperature dependence of *Q*_10_ values. *Q*_10_ values (open circles) were calculated from temperature jump experiments. A set of dotted lines indicates temperature-dependent *Q*_10_ values for fixed activation enthalpy (iso-enthalpy line). At higher temperatures (45 °C) the system behaves as if it was limited only by diffusion in bulk solution. (Reprinted with permission from Ref 106. Copyright 2009 The Rockefeller University Press)

This approach has been elegantly utilized by Kuno et al.^106^ to separate temperature effects on access and pore conductances in a quantitative study in rat microglia. They avoided changes in H^+^ concentration in any access region—a process likely to take seconds—by measuring the H^+^ current within a few milliseconds of a step change in temperature. Kuno et al.^106^ separated the apparent *Q*_10_ into a *Q*_10_ for the channel (2.8 at 5°C to 2.2 at 45°C) and a *Q*_10_ for the access resistance (about 1.2 at all temperatures tested). As Figure 10 shows the overall *Q*_10_ (open circles) was close to that of the channel itself only at low temperatures. At higher temperatures the system behaved as if it was limited only by the properties of the access pathway. Using these figures the activation enthalpy for the access resistance worked out to be 12.5 kJ/mol (3 kcal/mol) while that for the channel was the same as that for facilitated diffusion of glucose molecules (64 kJ/mol or 15.3 kcal.mol).

The combined *Q*_10_ reported by Kuno et al.^106^ is about 2.5 at 20°C which matches the data obtained on whole cells by DeCoursey and Cherny^103^(*Q*_10_ 2.1–3.1) as well as that obtained by Byerly and Suen^102^(*Q*_10_ 2.1). It also agrees reasonably well with data from wild type H_v_1 channels expressed in HEK-293 cells.^99^ Here the channel conductance in inside-out membrane patches had a *Q*_10_ of 3.3 (temperature range 9–32°C), somewhat lower than that for native channels in inside-out patches from alveolar epithelial cells (*Q*_10_ 5.3 below 20°C). Even lower values (*Q*_10_ 2.2) were obtained from modified H_v_1 channels which lacked the intracellular C-terminus and which are thought to exist in monomeric form. The differences in *Q*_10_ might suggest that the barrier to H^+^ conduction increases in channels that are constrained in some way either by their size or by being under tension in a membrane patch.

DeCoursey and Cherny^103^ provide a convenient summary of different activation energies that may be required by an H^+^ to access the channel and pass through it (their Table III). The activation energy of the access conductance obtained by Kuno et al.^106^ (3 kcal/mol) is close to the hydrogen bond strength in water while the activation energy for translocation through the cell membrane (15.3 kcal mol) matches that for hydrolysis (the deprotonation of water). Hydrolysis can account for the large flux of H^+^ across a phosphatidylcholine bilayer treated with the protonophore S-13^107^ and DeCoursey and Cherny^103^ have noted that if hydrolysis were rate limiting it might account for the near pH independence of the maximum proton channel conductance.

### Structure/Function Studies

The mouse, human, and *Ciona* proton channel clones have changed the way we think about H^+^ conduction. This is reflected in the name of the gene product chosen by the Okamura group; the voltage-sensor domain-only protein. Now Berger and Isacoff^108^ have found that the S4 segment's third arginine residue (position 211) sits at a constriction in the pathway. Replacing the arginine, which has a large positively charged guanidinium side-group, with the much smaller serine residue makes H_v_1 permeable to guanidinium ions. Berger and Isacoff suggest that arginine 211 together with the aspartate at a site mid-way along the S1 sequence (position 112) make up an H^+^ selectivity filter. It is evident that the two residues, one positive and one negative, are held apart by the rest of the channel structure otherwise the guanidinium ions would be unable to pass between them. Presumably the space is filled with water molecules.

By replacing aspartate 112 with a number of different amino acid residues Musset et al.^109^ have shed further light on the mechanism of selectivity. The only substitute that maintained H_v_1's H^+^ selectivity was glutamate. In many cases mutant channels became anion-selective. Even histidine failed to shuttle H^+^ as expected but instead produced a chloride (or methanesulfonate) permeable channel.

The critical role of the aspartate and arginine residues in determining selectivity implies that ions other than H^+^ have access to the heart of the native H_v_1 pathway. Presumably the negatively charged aspartate or glutamate repels anions while the positively charged arginine prevents the passage of the larger cations. Gaining access to more selective regions will depend on the properties of water in what is likely to be a confined space. The presence of ions like TEA^+^ can increase the viscosity of the local water while other smaller cations like Cs^+^ have a disruptive effect and reduce viscosity. TMA^+^ behaves more like a small cation^110^ indicating that the tolerances are quite fine. In *Lymnaea*^2^ the maximum H^+^ conductance was reduced by 33% when 55 mM Tris^+^ was substituted with an equivalent amount of TEA^+^ and it would be interesting to test whether TMA^+^ produces the same effect.

When specific arginine residues in the *Shaker* S4 voltage-sensor are replaced with histidine a proton pore is created in either the resting or activated state.^46^,^111^ Ramsey et al.^13^ have used amino acid substitutions to test whether H_v_1 is also a histidine-based conductance. H_v_1 contains 5 histidine residues in the transmembrane region; two of them seem to be accessible to the extracellular environment and two are expected to be accessible to the intracellular environment. When each one was separately converted to an alanine residue, the resulting changes in voltage-dependent current were so small as to make it unlikely that H^+^ permeation depends on a single histidine. Ramsey et al. suggest that in H_v_1 H^+^ is transferred not by direct interaction with specific amino acid side-chains but by hopping along a water wire. Supporting this mechanism are molecular dynamics simulations showing water molecules to be a more prominent feature within H_v_1 than in the voltage-sensing domains of potassium channels.^13^

As envisioned by Nagle and Morowitz,^12^ a hydrogen-bonded chain consisting of hydroxyls attached to sequence of polypeptide side groups is highly susceptible to disruption in that ‘a single break in the chain destroys its connectivity and conductivity’. In the absence of aspartate a chloride ion, which is not much larger than a water molecule, might simply fill a fault in the chain and travel with it across the membrane. However, if larger anions, like methanesulphonate, gain access to the heart of the proton channel they may disrupt the structure so much that a broad three-dimensional water-filled pore replaces the one-dimensional chain normally present.

The properties of water within a protein cavity, as opposed to water in bulk solution, are so little understood that it is not clear what the rate limiting step of H^+^ translocation would be. Cukierman^14^ describes a mechanism whereby H^+^ translocation is promoted by thermal fluctuations in the protein structure surrounding the water wire. Fluctuations that weaken or temporarily eliminate H-bonds between water and individual amino acid carbonyl groups allow the water molecules to move closer together and permit the transfer of H^+^ from water to water. This might explain why the apparent *Q*_10_ of native proton channels decreases at higher temperatures until it is only limited by the activation energy required to break hydrogen bonds. It may also go some way to explaining why the voltage-sensor-containing phosphatase in *Ciona* (Ci-VSP) generates gating currents but little or no steady current. Villalba-Galea et al.^112^ have shown that the presence of the phosphatase restricts the movement of the voltage sensor; it may stabilize the channel making it less subject to thermal agitation so that a complete water wire never forms.

## gp91-phox

By the time I met Lydia Henderson she and her colleagues Brian Chappell and Owen Jones^3^ had already established how human neutrophils generate superoxide, the free radical that kills off invading bacteria. They proposed that an electrogenic NADPH oxidase associated with the neutrophil cell membrane catalyzes the reduction of O_2_ to O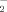 by converting NADPH to NADP^+^ and H^+^. Of the reaction products O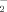 is released into the bathing medium but H^+^ remains in the cell cytoplasm and would alter the whole biochemistry of the cell unless allowed to escape. Henderson et al. suggested that the escape route was a ‘NADPH oxidase-associated H^+^ channel’ like the one that Roger Thomas and I had described in snail neurons. The idea was that the neutrophil would be depolarized by the flow of electrons during the generation of O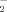; the depolarization would open up the proton channel and the efflux of H^+^ would minimize the acidification of the cytoplasm. The outward H^+^ current would also tend to repolarize the cell.

Analysis of the oxidase had shown that it was made up of a membrane-bound cytochrome consisting of gp91^phox^ and p22^phox^ together with the cytosolic proteins p47^phox^ and p67^phox^. H^+^ ion flux measurements on cell lines generated from individuals with chronic granulomatous disease, an inherited condition characterized by an inability to generate superoxide, lead to the idea that gp91^phox^ might be the H^+^ selective pathway. At a chance meeting Lydia Henderson told me she had expressed gp91^phox^ in Chinese hamster ovary (CHO) cells and demonstrated H^+^ fluxes in cells loaded with pH indicator. What she wanted to do next, she told me, was to learn how to patch-clamp the cells so that she could characterize the channels. I was happy to do what I could to help and was naturally delighted when Lydia found that the gp91^phox^-expressing cells generated large voltage-gated H^+^ selective currents. It then became evident that these currents could also be recorded from cells expressing only the NH_2_-terminal 230 amino acids. Subsequently we found that the histidine in position 115 seemed critically important for the selectivity of the pathway while histidines in positions 111 and 119 appeared to play a role in gating.^47^

During the course of this work Lydia accepted an invitation from Tom DeCoursey to visit his laboratory in Chicago. Upon her return she told me that the cell lines had not traveled well and for some reason had not performed as expected. All was not lost however, because during the course of their experiments she, Cherny and DeCoursey^113^ found that even non-transfected CHO cells exhibited a small H^+^ current. The average current was about 10 pA in non-transfected cells (pH_o_ 7.4; pH_i_ 5.5; membrane potential +40 mV) which was about 1% of the value that Lydia & I had been getting in the gp91^phox^-expressing cells. In 2001 Lydia's findings were confirmed by Maturana et al. 114 working in Nicolas Demaurex's laboratory in Geneva. These authors used mainly human embryonic kidney 293 cells some of which exhibited H^+^ currents in a non-transfected state just like the CHO cells did.

At this stage the evidence that gp91^phox^ was the proton channel was entirely circumstantial. The challenge was to find voltage-gated H^+^ currents in the absence of membrane-bound gp91^phox^. There were reports that gp91^phox^-free knock-out mice had neutrophils with H^+^ currents but we questioned whether the critical experiment had yet been done. Our reasons were: (1) it was evident that the human genome contained more than one gene encoding a gp91^phox^-like protein, (2) these gp91^phox^ look-alikes might also be proton channels, (3) in knock-out animals the gp91^phox^ look-alikes might be up-regulated. It was unfortunate that the available antibodies appeared to bind to a number of gp91^phox^ look-alikes. The arguments were well-covered in previous commentaries.^115^,^116^

DeCoursey et al.^116^ were not convinced by our arguments. They concluded that the proton channel was probably not gp91^phox^ but that gp91^phox^ might modulate the activity of a pre-existing proton channel. Other workers suggested that assembly of the oxidase was necessary to activate a closely associated but separate H^+^ conducting entity.^117^ In 2001 Tom sent me a copy of a paper titled: ‘The gp91^phox^ component of NADPH oxidase is not the voltage-gated proton channel in phagocytes, but it helps.’^118^ He added the comment: ‘Will be fun to look back in 10 years when we “know” what the real story is'. Ten years later the story is, indeed, a little clearer. El Chemaly et al.^119^ in Nicolas Demaurex's laboratory have established that neutrophils from mice bearing a targeted disrupting mutation in the VSOP/H_v_1 gene (VSOP/H_v_1-/- mice^120^) lack H^+^ currents. They have normal electron currents, showing that their oxidase is fully functional, but they cannot conduct H^+^. It seems clear that the VSOP/H_v_1 gene product is necessary for H^+^ conduction.

To exclude the possibility that H^+^ conduction by H_v_1 requires the presence of an as yet unknown protein, Lee, Letts and MacKinnon^121^ have reconstituted purified H_v_1 channels into synthetic lipid liposomes and demonstrated an inward flux of H^+^ under an appropriate electrochemical gradient. As a control the voltage-gated potassium channel from the archeabacterium *Aeropyrum pernix* (K_v_AP) was tested and found to be unable to conduct H^+^ under the same conditions. In fact ‘native’ proton channels should not conduct H^+^ under these conditions either. The only reason why H_v_1 works this way is that its voltage-dependence is shifted by about −30 mV compared with native mammalian proton channels.^122^ The reason for the shift is not known but it seems possible that native cells contain additional co-factors.

The promotion of H^+^ currents in gp91^phox^-transfected cells is still a puzzle. In particular the precise role of the histidine at position 115 remains unexplained. In activated neutrophils there is a puzzling correlation between the activity of the NADPH oxidase (judged from the electron current) and the shift in the voltage-dependence of the proton channel.^123^ It may be that gp91^phox^ promotes proton channel assembly or alternatively that titration of histidine 115 plays a key role in tranferring H^+^ to the channel. Histidine has an imidazole side chain with two nitrogens, one slightly acidic, the other basic, which can both donate and accept H^+^ and which could therefore shuttle H^+^ towards and away from active sites.

Although histidine-based H^+^ transfer is well known to occur across the omega pore of the *Shaker* K channel^45^ and through the pore formed by the influenza A virus,^124^ it is time to put the gp91^phox^ hypothesis to bed. However it would be surprising if Onsager's ‘hydrogen bonded chain’ existed in only one form. Any thread of water molecules that stretches across the cell membrane should provide an ionic pathway. The trick is to establish its selectivity and ensure that its operating range is appropriate to its physiological function. All that is required for H^+^ selectivity apparently, is a constriction with a single acidic residue, or an appropriately placed histidine residue. Achieving an appropriate voltage range for gating seems far more demanding. It appears to require contributions from the entire molecule^125^ as well as auxiliary structures—perhaps even including gp91^phox^.

## POSTSCRIPT

It is worth underlining the importance of comparative studies in the development of the proton channel field. During the evolution of the enormously varied life-forms present on our planet, natural selection has adjusted the structure of individual proteins to perform an astonishing number of different functions. Exploring the relationship between these functions and the variations in protein structure on which they depend is an architectural delight especially for anyone who enjoys the unforeseen. Surprise is an essential by-product of the progress made when observation shows up the inadequacy of our metaphors. It is natural for research to cluster around a limited number of the planet's life forms; perhaps for convenience or perhaps because of the sheer amount of accreted information. But if you want a new perspective, if you want the unforeseen, try searching out neglected life forms, for that is often where the surprises are situated.^126^

Questions of utility do persist, however. How can I wrest some clear practical benefit from the unfashionable end of the life-form spectrum? Is my obsession with this protein or that species just too self-indulgent? Perhaps they are obscure variants with no relevance to the ‘real world’ of medicine and commerce. The existence of H^+^-based action potentials surprised many of us but even workers in the field must have wondered whether they were limited to the dinoflagellates. I wish I had known about the *Noctiluca* research earlier. Any evidence for the widespread distribution of proton channels would have been welcome. Unfortunately, I was not looking in the right places; literature searches were rather hit and miss in the days before the internet. Consequently it was left to Lydia Henderson and Tom DeCoursey to spike general interest by providing proton channels with a human-based function.

In fact those of us who worked with snail neurons chose them for convenience rather than for any other reason. We did not want too many surprises; the whole point was that they were to be a model for neurons in higher animals. We needed to show that snail neurons behaved like mammalian nerve cells before we could move on to more challenging areas. For some of us the cell cytoplasm was the new frontier for neuroscience. In the days before the whole-cell patch clamp and the confocal microscope the only way to explore the cell cytosol was to use the largest nerve cells available. Snail nerve cells were huge and what is more, in skilled hands they were strong enough to survive penetration with blunt electrodes, repeated pressure injection and long periods of intracellular perfusion.

If the roots of our understanding of proton channel function can be traced to research on mammals, axolotls, snails, and dinoflagelates, our understanding of proton channel structure has benefitted from the insight of those who utilized the giant chromosomes of *Drosophila*, the simplicity and stability of certain bacterial proteins and those who recognized the subtle clues provided by the sea squirt *Ciona*. Genome research has made comparative biology fashionable again and has provided us with the tools to seek out the enormous range of functions that proton channel structures are adapted to perform.
